# Optimal sizing of renewable generation and storage for grid-connected CO_2_ electrolysis: a Dutch case study on syngas production for e-SAF

**DOI:** 10.1039/d6gc04304f

**Published:** 2026-08-19

**Authors:** Thijmen Wiltink, Andrea Ramírez, Mar Pérez-Fortes

**Affiliations:** a Department of Engineering, Systems, and Services, Faculty of Technology, Policy, and Management, Delft University of Technology Jaffalaan 5 2628 BX Delft Netherlands M.D.M.Perez-Fortes@tudelft.nl; b Department of Chemical Engineering, Faculty of Applied Sciences, Delft University of Technology Van der Maasweg 9 2629 HZ Delft Netherlands

## Abstract

Co-electrolysis of CO_2_ (CO_2_E) and water enables the production of syngas for sustainable aviation fuels (SAF) that are compliant with European RFNBO (renewable fuels of non-biological origin) regulations. However, a mismatch exists between the intermittent renewable electricity supply and the continuous operation of the downstream Fischer–Tropsch plants. To address this, we developed a two-stage linear optimization model to optimize the operation of a 540 MW electrolysis plant, alongside the sizing and operation of the connected renewable generation, battery storage, and syngas storage. Applying this model to a Dutch case study, we explored grid integration with an electrolyzer across future scenarios with global warming potentials (GWPs) ranging between 35 and 370 g CO_2_-eq per kWh. For generation, the preferred renewable mix is onshore wind combined with PV. When grid mix electricity consumption is restricted, the electrolyzer has an optimal capacity factor of 78% but requires a battery of comparable capacity to the electrolyzer and multi-kilotonne syngas storage to ensure continuous output. Crucially, we found that producing RFNBO-compliant syngas for SAF is impossible with the 2025 Dutch grid mix. Even at a reduced grid intensity of 205 g CO_2_-eq per kWh, RFNBO compliance limits grid consumption to just 1% of grid mix electricity per hour. This results in a levelized cost of 2350 EUR_2019_ per tonne syngas. Unrestricted grid electricity consumption becomes feasible when emissions drop below 36 g CO_2_-eq per kWh, reducing production costs by 43% (1344 EUR_2019_ per tonne syngas). Consequently, we demonstrate that grid composition intensity is a bottleneck for the short-term economic viability and regulatory compliance of CO_2_E-based SAF in the Netherlands.

## Abstract 2

Co-electrolysis enables RFNBO-compliant sustainable aviation fuels, but grid carbon intensity is a major bottleneck. Our optimization model shows that robust storage and greener grids are essential for economically viable SAF production.

## Graphical abstract

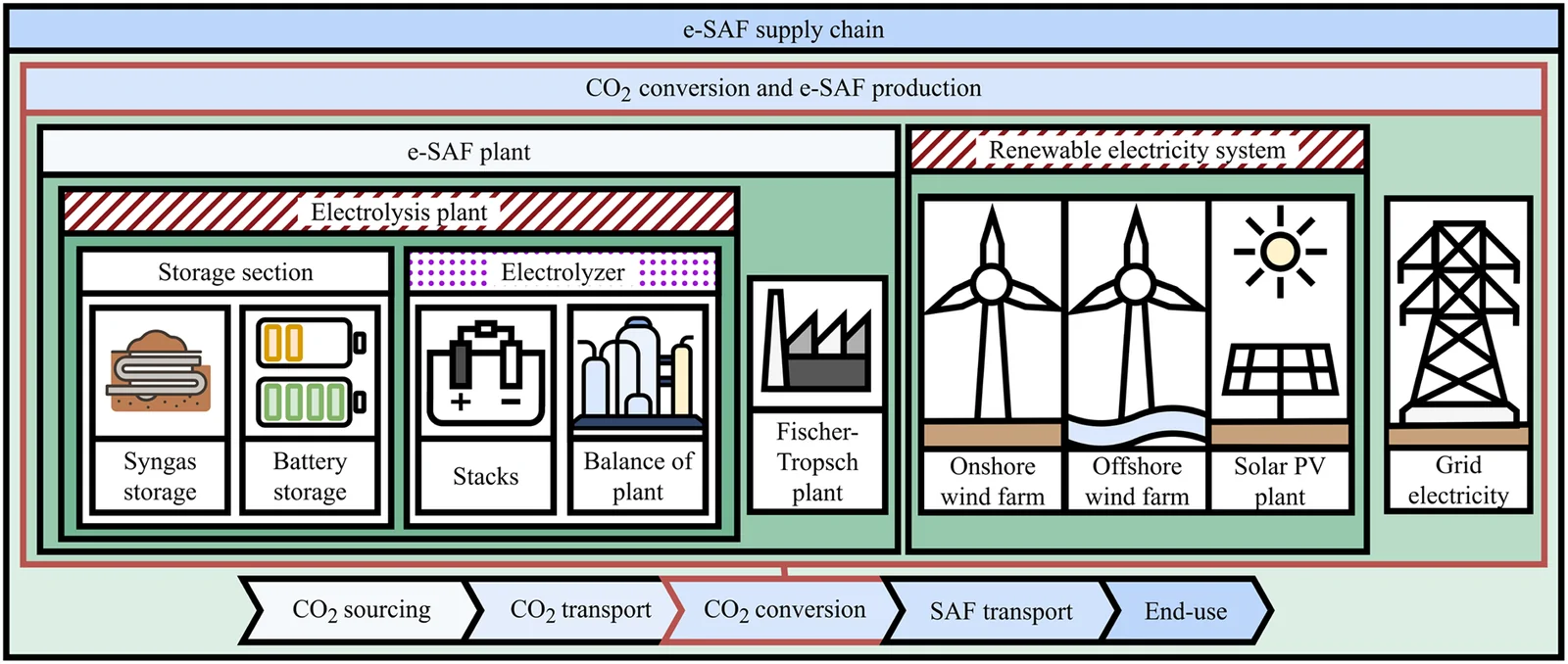

**Green foundation**

1. This work introduces an optimization framework for the sizing and operation of a CO_2_ electrolyzer within the current industrial context. By integrating renewable electricity and CO_2_ feedstocks, the study contributes to the defossilization of the conventional chemical and fuel industries.

2. Qualitatively, this work demonstrates the role of a CO_2_ electrolyzer in the e-SAF supply chain, identifying financial and practical bottlenecks. Moreover, it provides clear direction on how a CO_2_ electrolyzer should be operated in an industrial setting, specifically addressing the need for green electricity grids and storage systems.

3. The insights from this study could be applied across diverse geographical contexts to identify locations where CO_2_ electrolyzers are most environmentally and economically competitive. This would facilitate the identification of high-profile demonstration sites. Furthermore, the framework could be extended to include environmental impact categories beyond global warming potential (GWP).

## 1 Introduction

The aviation sector is slow to decarbonize due to its near-exclusive reliance on fossil fuels. In 2022, sustainable aviation fuels (SAF) accounted for only 0.1% of the total jet fuel production.^1^ In the EU, replacing fossil fuels with greener alternatives is considered the main strategy for this sector to meet the decarbonization ambitions.^2^ For instance, the ReFuelEU Aviation regulation puts forward a 6% SAF obligation in 2030 to accelerate SAF production (2023/2405/EC). This includes a 1.2% sub-target specific to synthetic fuels or e-SAF, averaged over 2030 and 2031. Synthetic fuels are defined as being produced *via* chemical synthesis using renewable electricity. Therefore, to guarantee their environmental sustainability, synthetic fuels must meet strict criteria as specified in the certification requirements for renewable fuels of non-biological origin (RFNBO).^3^ According to the RFNBO regulation, the CO_2_ electrolyzer stack needs to be fed with carbon-neutral electricity (which fulfills conditions on additionality, temporal correlation, and geographic correlation (2023/1184/EC)). An exception is made when the grid mix emissions are below 64.8 g CO_2_-eq per kWh (or 18 CO_2_-eq per MJ). The balance of plant of the electrolyzer and the background system (*i.e.*, CO_2_ sourcing, transport conditioning, SAF transport, and end-use) can be fed with grid mix electricity,^4^ but the emissions from consuming this electricity are allocated to the SAF. Across the entire SAF supply chain (SC), at least a 70% global warming potential (GWP) reduction must be achieved compared to a fossil benchmark. RFNBO certification for SAF is not only essential for contributing to emission reductions and legally binding targets but also enables producers to command a premium for the end-product.

Co-electrolysis of CO_2_ (CO_2_E) and water is part of the portfolio of technologies that have the potential to produce intermediate compounds for the synthesis of fuels that could meet the RFNBO certification criteria.^5^ Through CO_2_ co-electrolysis, syngas can be produced from CO_2_, electricity, and water. Syngas, a mixture of carbon monoxide (CO) and hydrogen (H_2_), can be converted into larger hydrocarbons and chemicals *via* Fischer–Tropsch (FT) or methanol synthesis.^6^ In the current industrial practice, these processes typically operate continuously, with installed capacities used for more than 90% per year under steady-state conditions.^7^ An electrolysis plant operates between the steady-state production of fuels/chemicals and the intermittent electricity supply from renewables.^8^ This plant must accommodate fluctuations in electricity input while ensuring a stable product output, which requires a high degree of operational flexibility.^9^ Achieving this cost-effectively depends on the appropriate selection of units and sizing and operational strategies.^10^ Optimization models can be used to systematically design and plan the operation of an electrolysis-based plant.^11^ Drawing primarily from the hydrogen sector, the literature identifies the following strategies to deal with the challenge of fluctuating electricity supply and continuous product demand:

i.The use of batteries enables the storage of energy from renewable energy sources (RES) in periods when electricity is not immediately needed.^12^ This could allow a reduction in the size of the electrolysis plant compared to the renewable energy plant.

ii. Oversizing the renewable energy generation capacity in relation to the electrolyzer capacity results in a greater availability of renewable electricity. Also, the surplus electricity will be larger, which can be managed through curtailment, electricity selling or battery storage.^13^

iii. The storage of an intermediate allows for temporal decoupling of production and final use.^14^

iv. The use of a mixed profile of renewables:^15,16^ photovoltaics (PV) and wind can complement each other since their availability is weakly anticorrelated.^17^

v. Connection to the grid: in moments when the electricity price is low or there is little RES production, the grid can supply the electricity needed to fulfil the production requirements.^18^

In the regulations, while the electrolyzer stack is strictly bound to renewable electricity sourcing, the balance of plant is allowed to consume grid mix electricity, albeit restricted by the RFNBO emission reduction threshold of 70% (2023/1184/EC). Since the consumption of grid mix electricity carries greenhouse gas (GHG) emissions, the fraction of grid mix electricity that can be consumed to comply with the RFNBO regulation depends on the grid's carbon footprint.^4^ Simultaneously, the grid mix emissions also impact the GWP of the background system. With the electricity grids rapidly decarbonizing,^19^ the amount of grid mix electricity that can be consumed in the electrolysis-based plant will increase over time. It is, however, unknown how the evolution of allowed grid mix consumption would affect the sizing and scheduling strategies required to produce RFNBO-compliant syngas at the lowest cost. The current paper aims to address this gap by optimizing the configuration of the electricity supply, grid electricity consumption and storage. It optimizes the sizing strategy and CO_2_ electrolyzer operation to minimize costs, while the operation is constrained by evolving grid mix-consumption limits.

## 2 Case study description

**Fig. 1:**
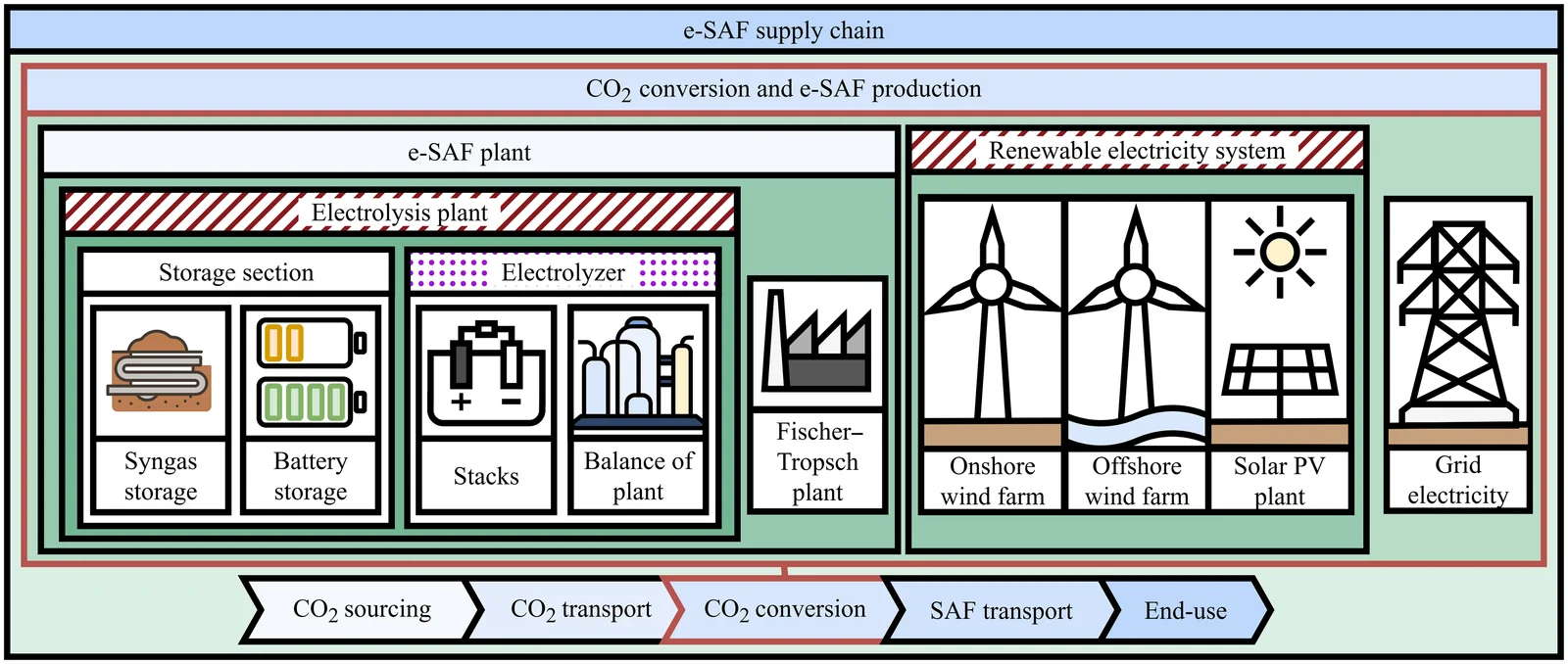
Visualization of the case study and used terminology throughout the work. The hatched blocks are considered the foreground system and part of the optimization model, with the dotted background having a fixed input size. The solid blocks are part of the background system and count towards the greenhouse gas emissions of the supply chain.

The case study investigates the sizing and operational decisions of a CO_2_-to-syngas electrolysis plant producing RFNBO-compliant syngas, see Fig. 1. In the optimization, the electrolysis plant and renewable electricity system are optimized around a fixed input electrolyzer size of 540 MW. The syngas is supplied to a FT plant, which converts it into e-SAF. The FT plant section of the e-SAF plant is not part of the optimization and is considered part of the background system. The case study is analysed from the perspective of the syngas producer, whose primary objective is to minimize the annual costs (CAPEX and OPEX) to produce RFNBO-compliant syngas. The secondary objective is to use all installed equipment to its fullest potential by optimizing the operation of the electrolysis plant. It was assumed that the project was announced in 2025 and would be fully operational by 2030. To power the electrolysis plant, the syngas producer invests in a mixed electricity profile of solar PV, onshore wind, and offshore wind. Finally, it was assumed that the syngas producer was the only investor in the electrolysis plant and renewable electricity plants.

### 2.1 Geographic scope of the case study

The electrolysis plant was assumed to be located in a chemical cluster in the Botlek area in Rotterdam, the Netherlands. The Rotterdam location offers a strategic advantage due to the potential availability of biogenic CO_2_; there is a bioethanol plant in the vicinity which could provide 220 kt per year of biogenic CO_2_.^20^ Moreover, the development of the carbon capture and storage (CCS) project Porthos is expected to accelerate the CCS-related infrastructure implementation.^21^

### 2.2 CO_2_ sourcing and CO_2_ transport

**Fig. 2:**
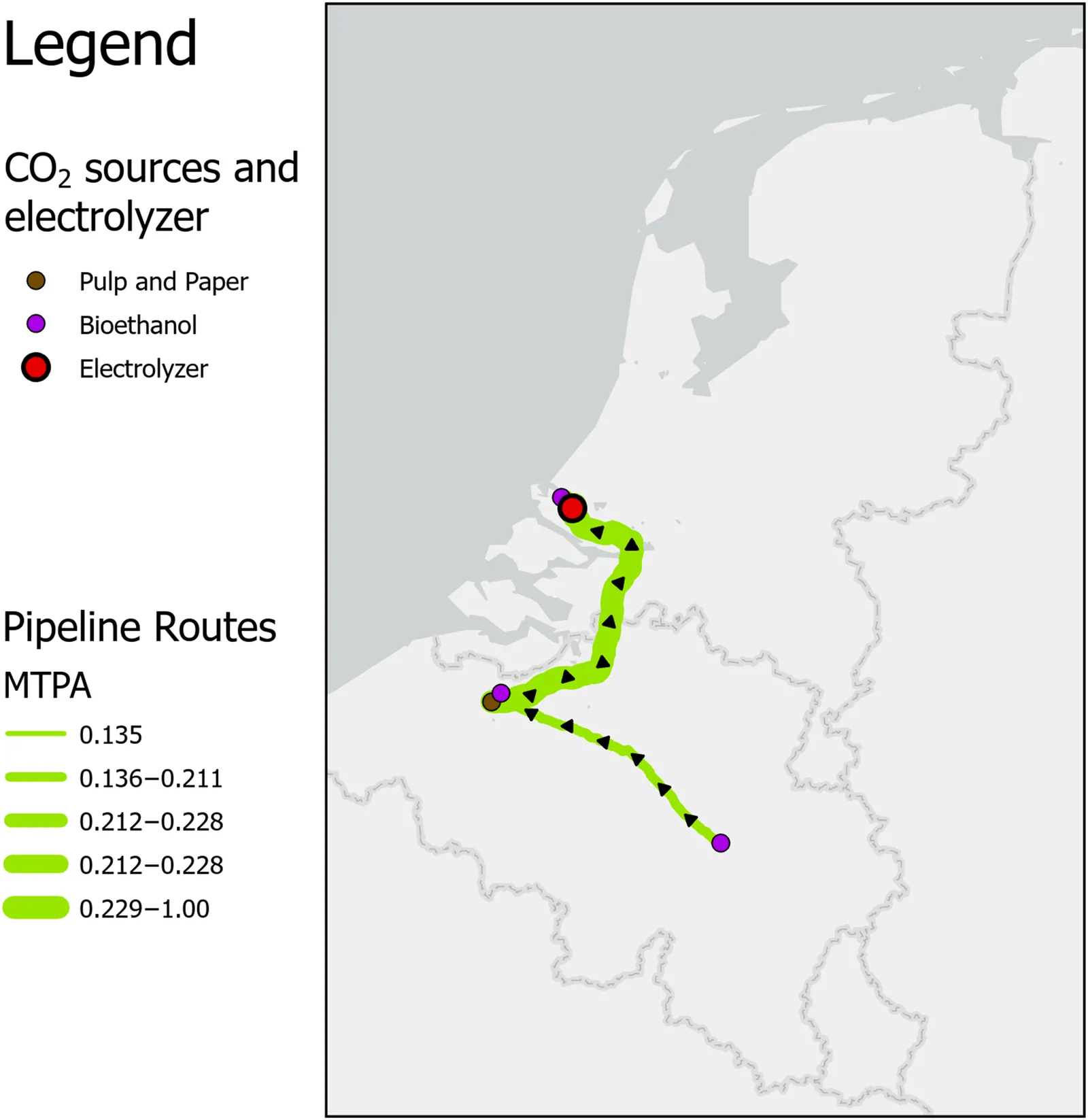
Biogenic CO_2_ sources and pipeline design. MTPA: Mtonnes per annum.

The plant was assumed to receive biogenic CO_2_ in Belgium and the Netherlands *via* a dedicated pipeline network, as depicted in Fig. 2. This design was developed using the supply chain optimization tool from previous work.^22,23^ However, the geographic scope was adapted to fit the Netherlands and Belgium specifically. Furthermore, only CO_2_ sources which comply with the RFNBO regulation were included, namely pulp and paper and bioethanol.

Current plans include the construction of a CO_2_ pipeline between Antwerp and Rotterdam as part of the Delta Schelde CO_2_ project.^24^ However, this pipeline is intended primarily to transport fossil CO_2_ for storage. To comply with the RFNBO regulation, fossil CO_2_ is expected to remain separate from biogenic CO_2_. Therefore separate infrastructure will be necessary.

The infrastructure was designed to deliver 1.0 Mtonne of CO_2_ per year to the electrolyzer location. It was assumed that the plant could receive any amount within the 1.0 Mtonne range of CO_2_ at any time. When the electrolyzer is not operating, the CO_2_ is sent to permanent storage, *via* a satellite pipeline connection to Porthos (which is not shown in Fig. 2 and not further considered in this work). The satellite pipeline was sized independently of the CO_2_ consumption in the electrolyzer. The CO_2_ was supplied at a constant price of 75 EUR_2019_ per tonne. However, securing sufficient quantities of biogenic CO_2_ presents a supply-side risk, and the required capture and transport infrastructure is still under development; therefore, future price certainty cannot be guaranteed. This price was the result of running our previously developed supply chain design model,^22,23^ which calculates the CO_2_ cost of the given network. The CO_2_ price, which resulted from this optimization, was input for the model used in this work.

### 2.3 Electrolyzer

The electrolyzer capacity was fixed at 540 MW. This includes 415 MW of electrolyzer stacks and 125 MW for the balance of plant. At maximum production, the plant consumes approximately 575 kilotonnes of CO_2_ and 487 kilotonnes of water per year. These feedstock flows correspond to a syngas production of 420 kilotonnes per year, which in turn can yield 146 kilotonnes of SAF (based on the yield factors of Boilley *et al.*^25^). This syngas output of the electrolysis plant aligned well with the typical small-scale size of a FT plant.^26,27^ The 540 MW capacity was based on the design sizes of Noordende *et al.*^28^ They assumed that a 90 MW system can be numbered up to reach larger sizes. This system consisted of ten individual modules, each with a capacity of 9 MW, which contained balance of plant equipment (*e.g.*, e-heaters and syngas compression) and power electronics. Within each module, there were six 1.5 MW hot boxes that could ramp between 32.5% and 100% capacity.^29^ In total, the electrolyzer consisted of 360 individual hotboxes that could individually ramp up and down their load. Furthermore, the hot box could not ramp below 32.5%. Then the hot box needed to enter the hot standby mode, consuming still about 7% of electricity without producing syngas.^30^ By using hot standby and individual modules, the plant as a whole could ramp continuously between 7% and 100%. Such a method of operation has been shown in the literature to operate feasibly under realistic RES power variability.^31^

### 2.4 Storage section

The electrolysis plant could invest in both electricity and syngas storage. The model assumed the use of lithium-ion batteries without a maximum capacity for energy storage. This is justified by their modular nature and recent large-scale announcements of 300 and 350 MW battery projects in the Netherlands, set to be operational by 2027.^32,33^ For syngas, underground pipeline bulk storage was selected. This technology is capable of storing in the required kiloton range and does not depend on the availability of specific geological formations, unlike other bulk storage options such as salt and lined rock caverns.^34,35^

### 2.5 Syngas supply to a FT plant

The electrolysis plant was assumed to be located close to a hypothetical SAF producer within the chemical cluster. Carbon dioxide and electricity were transported to the plant site and the intermediate syngas was assumed to be transported over a negligible distance to its end-use location.

The electrolysis plant needed to supply syngas with only limited fluctuations to interface with a conventional FT plant. We assumed that the hourly flow could vary between 70% and 100% of its nominal capacity^36^ and that the FT plant cannot be turned off, with an yearly average of 91.3% (equivalent to 8000 full load operating hours). It is assumed that the electrolysis plant produces syngas whose output stream matches the intake requirements of the FT plant. In this way, the demand is always assumed to be met each hour. Furthermore, the size of the FT plant was not part of the optimization and assumed to match the produced syngas flow.

### 2.6 Renewable electricity system

The maximum size for a PV farm was set to 175 MW, which is in the same order of magnitude as the largest planned PV park in the Netherlands.^37^ Onshore wind was capped at 515 MW capacity.^38^ No maximum capacity was enforced for offshore wind. There is currently 5.5 GW of offshore wind under construction, with an additional 11.5 GW planned in the near future (2030).^39^ The renewable electricity production profiles were based on six years of input electricity data at an hourly resolution (2019–2024), with hourly average renewable energy capacity factors (CF) from the Netherlands.^40^

### 2.7 Grid electricity supply

The electrolyzer and renewable energy sources were grid-connected without a direct transmission line between generation and the electrolysis plant, *i.e.*, the plant received all its electricity *via* the grid. A grid fee for receiving electricity of 18 EUR_2019_ per MWh^41^ was charged; however, when consuming self-generated electricity, only 90% of the fee needed to be paid. When selling back electricity to the grid, no feed-in tariff was considered, aligning with the current regulation.^42^ For the grid electricity costs, the ENTSO-E day-ahead market prices were used over the 2019–2024 time horizon.^43^ Potential grid congestion issues were not considered in this study.

## 3 Method

### 3.1 Modeling approach: a two-stage linear program

**Fig. 3:**
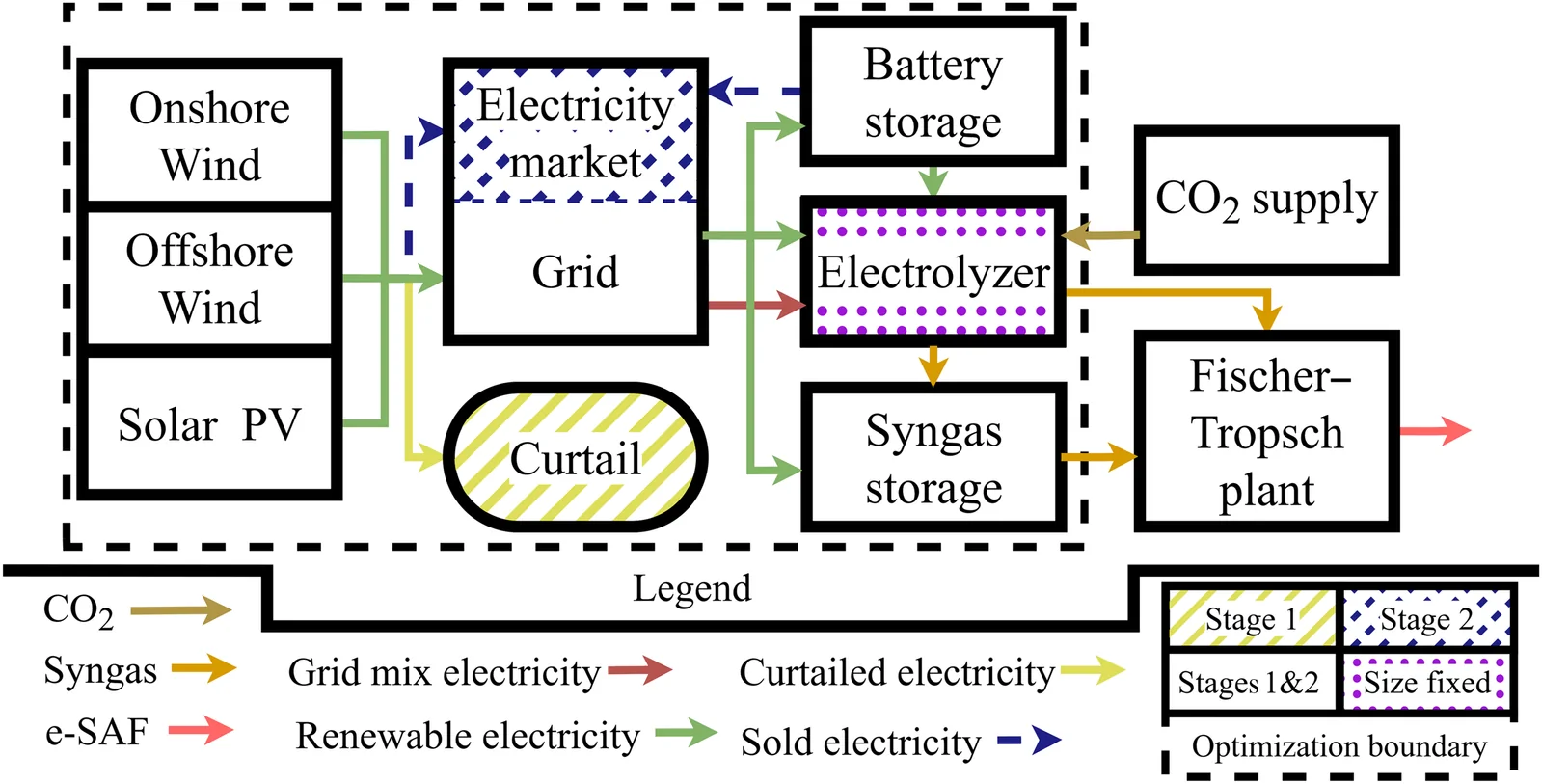
Two-stage sizing and operation optimization problem of the CO_2_ electrolysis plant and renewable electricity system. The elements within the dashed boundary are considered in the optimization framework. The white background elements are optimized in both stages, while curtailment is only considered in the first stage and selling electricity from the grid only performed in the second stage. The electrolyzer size is fixed at 540 MW, and only the operation is optimized. Note that water is not shown as feedstock for the co-electrolysis as it is not explicitly treated in this work.

The model was developed from scratch as a two-stage linear and mixed integer optimization. The product (syngas), input and electricity flows are shown in Fig. 3. The dashed line represents the optimization boundary in which the size and operation of the included elements are optimized. In the first stage, the model determined the lowest cost to meet a set syngas production target given a fixed electrolyzer size. The model was optimized for varying syngas targets. By changing the target, the optimal syngas production amount that results in the lowest production cost given a fixed electrolyzer size was found.

The decision variables were the sizes of the electricity supply (PV and/or wind) and storage section (batteries and/or syngas). Also, the amount of renewable energy curtailment was sized. In the second stage, the size of the equipment was fixed, and the system's operation was optimized. In this stage, curtailed electricity could be sold to the grid, and the battery could be used to store and sell renewable electricity at favorable moments. The additional revenues from selling electricity were subtracted from the costs resulting in the net annualized cost of syngas production. Since all the generation costs were allocated to the syngas production cost, all the revenues from electricity selling were also allocated to the syngas production. This two-stage approach is crucial as it decouples strategic sizing decisions from operational decisions. Furthermore, it allows to split the main and secondary objectives: namely, the main objective was producing syngas for the lowest cost with the smallest investment and the secondary objective was getting the most revenue out of the installed equipment. This aligned with the primary goal of being a syngas producer instead of a renewable energy producer.

The electrolyzer has the option to consume electricity from the grid mix. This consumption percentage was restricted by the grid's GWP. Five different grid carbon footprints were evaluated based on future scenarios from 2025 to 2045 in the Netherlands, which are elaborated in section 3.2. The percentage of grid mix electricity that could be consumed results from calculating the GWP of the complete e-SAF supply chain, see section 4.1.

The model assumed perfect operation, where compliance conditions were always met. Since the project is scheduled to be operational in 2030, compliance needs to be demonstrated on a per-hour basis (EC/2023/1184). In practice, missing a single requirement in one hour due to unfavorable conditions disqualifies the product for fuel applications in that hour. Therefore, a syngas producer would likely seek a second offtaker for a chemical application, which are not bound by the RFNBO certification regulations. In such a case, the syngas producer can still sell any non-compliant syngas. This hedges against the risk of producing a valuable product that cannot be sold.

The first stage was solved as an LP (linear programming) problem. In the second stage, binary variables were introduced to account for operational constraints (*e.g.*, simultaneous (un)loading or (dis)charging). This resulted in a MILP (mixed integer linear programming) formulation. The full mathematical description can be found in the SI, see Mathematical formulation. The model contains 2.1 million equations and 1.6 million variables and was implemented in GAMS (General Algebraic Modeling System) (version 49). Gurboi 12.0 was used to solve the optimization on the DelftBlue supercomputer.^44^ Here, it ran on 12 cores with 48 GB of memory available. On average, the different runs solved to optimality in just below 4 hours. The longest run time was just above 8 hours.

### 3.2 Grid global warming potential scenarios

The grid GWP determines the maximum grid mix electricity percentage which is allowed in each hour. This percentage served as an input into the model based on different future scenarios, summarized in Table 1. Since all electricity is supplied *via* the grid, only the consumption from the grid mix was constraint *via* this percentage. The model was solved as independent scenarios by changing this upper limit.

**Table 1:** Assumptions for grid global warming potential (GWP) and allowed consumption limits (2025–2045)

Scenario no.	Year	Scenario name^a^	Limiting value	Unit	Note
1	2025	2025-370	370	g CO_2_-eq per kWh	European Environment Agency^19^
2	2030	2030-205	205	g CO_2_-eq per kWh	Adriaansens and Jetten^41^
3	2035	2035-138	138	g CO_2_-eq per kWh	Adriaansens and Jetten^41^
4	2040	2040-65	65	g CO_2_-eq per kWh	Derived from EC/2023/1184^b^
5	2045	2045-100%	100	% grid mix	Assumed

a Scenario names denote [year]-[limiting value].

b Value derived from the threshold of 18 g CO_2_-eq per MJ (EC/2023/1184).

Grid emission projections were based on scenarios specific to the Netherlands for the period 2025–2045, resulting in 5 scenarios. The 2025 grid mix electricity percentage was calculated based on the 2025 Dutch grid emissions.^19^ The 2030 and 2035 scenarios were based on grid mix emission projections communicated to the Dutch House of Representatives^41^ and set to 205 and 138 kg CO_2_ equivalents per MWh, respectively. Following this trend, the 2040 scenario assumed that the Dutch electricity grid mix would meet the threshold for lifting the renewable energy requirement for RFNBO at 18 g CO_2_-eq per MJ (EC/2023/1184). From this point onward, grid mix electricity would count as renewable. This designation implies that the restrictions on the type of electricity, which need to be consumed in the electrolyzer stacks, are lifted. However, it is not a given that with these grid mix emissions, the product would also meet the 70% RFNBO emission reduction target when utilizing only grid electricity. Therefore, in a 2045 scenario, it was assumed that the grid mix emissions were low enough that all the electricity could be supplied from the mix, thereby meeting both the electricity and the GWP requirements. The GWPs from scenarios 1–4 in Table 1 limit the grid mix electricity consumption, with unlimited access in scenario 5.

### 3.3 Model description

#### Objective function: (net) annualized cost minimization

Throughout the description of the key equations, variables are denoted in lowercase italics and parameters in capital italics. The variable domains are indicated using italic subscripts, and roman superscripts provide additional details about the symbols. In the first stage, the objective function (eqn (1)) aimed to minimize the annualized cost *z*^St1^_sgt,syr_ in EUR_2019_ per year while satisfying a syngas production target (sgt) in ktonnes per year for every scenario considered. Set scn is the scenario number from Table 1, which corresponds to the maximum grid mix consumption percentage.

**eqObj**_**sgt,scn**_

Let SGT = {150, 160, …, 420} and SCN = {1, …, 5}1
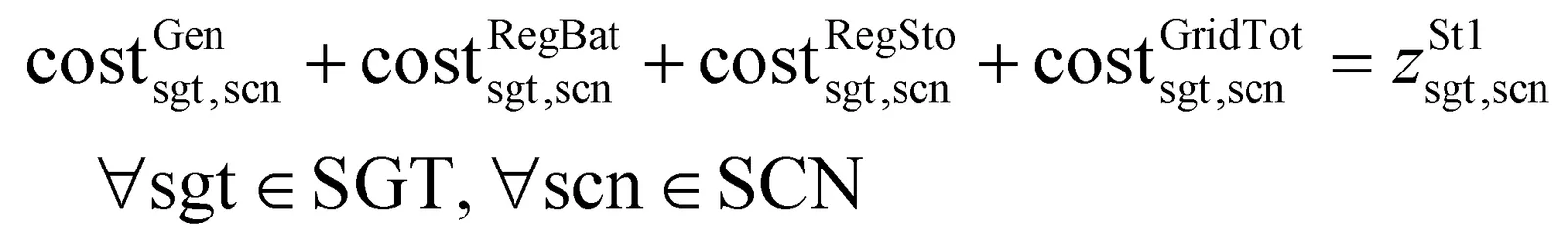


Eqn (1) consists of four cost categories: (i) generation (cost^Gen^), (ii) battery (cost^RegBat^), (iii) syngas storage (cost^RegSto^), and (iv) grid electricity (cost^GridTot^). The cost categories involve the annualized cost of CAPEX and OPEX. Since the size of the electrolyzer was fixed, it was not part of the objective function.

In the second stage, the system operation was re-optimized with the capacities resulting from the first stage of the equipment fixed. The stage 2 objective function considered the revenues generated from selling electricity (rev^Elec^_*t*,sgt,scn_), subtracted from the objective variable (*z*^St1^_sgt,scn_) of the first stage (eqn (3)), resulting in the net cost of production (*z*^St2^_sgt,scn_). At any hour in set *T*, the electricity sold directly from generation (*e*^Sell^_*t*,sgt,scn_) and the electricity discharged from the battery and sold in the grid (*e*^DC_Sell^_*t*,sgt,scn_) were determined. These amounts could be sold for the day-ahead electricity price (ELEC^Price^_*t*_) (eqn (2)).^43^ Set *T* contained 52 608 hourly data points spanning over six years (YR), from 1 January 2019 (00 : 00) to 31 December 2024 (23 : 00). The revenues and operating expenses (OPEX) were summed over the entire time horizon and averaged on an annual basis to align with the annualized CAPEX. In this way, long-term operational variability is considered in the strategic sizing decision.

**eqSell**_***t*,sgt,scn**_

Let *T* = {1, …, 52 608}2
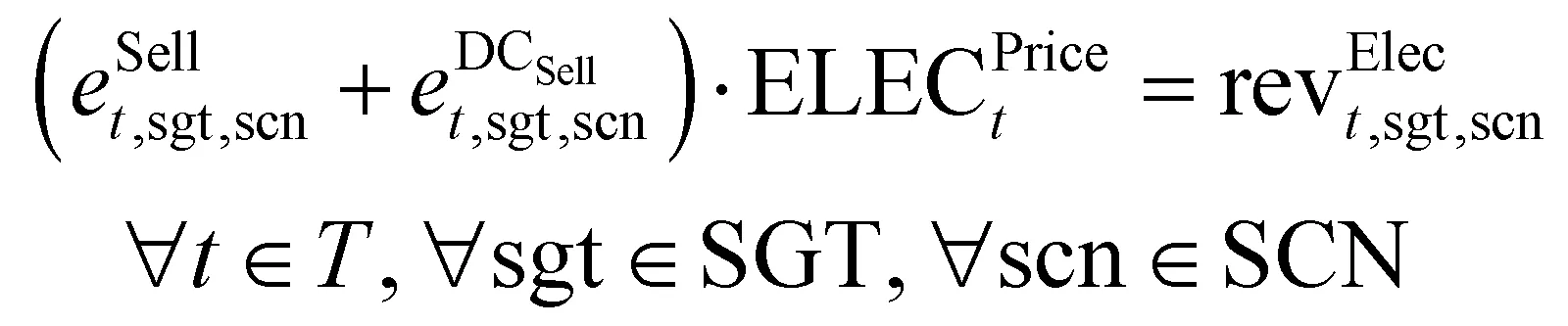


**eqObj2**_**sgt,scn**_

3

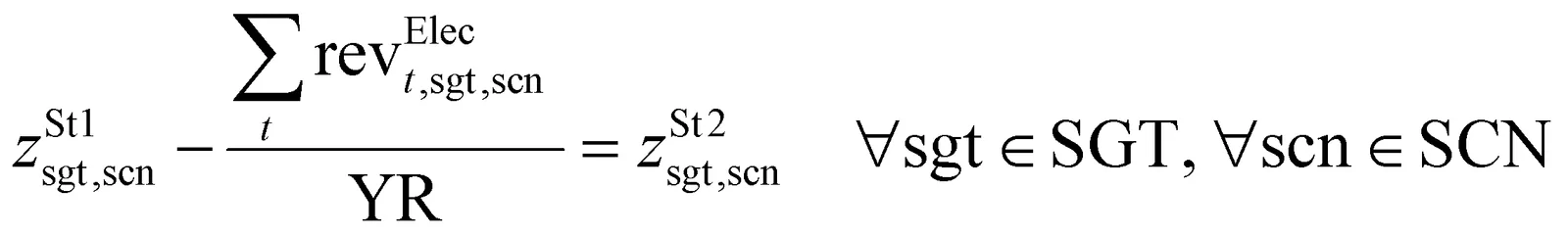




After the second stage optimization, the net levelized cost of production of syngas (LCOSG_sgt,scn_) was calculated considering the syngas production target (SG^Target^_sgt,scn_), as in eqn (4). Also, the regularizers needed to be subtracted, which are described in more detail in the Battery section.

**eqLev**_**sgt,scn**_

4

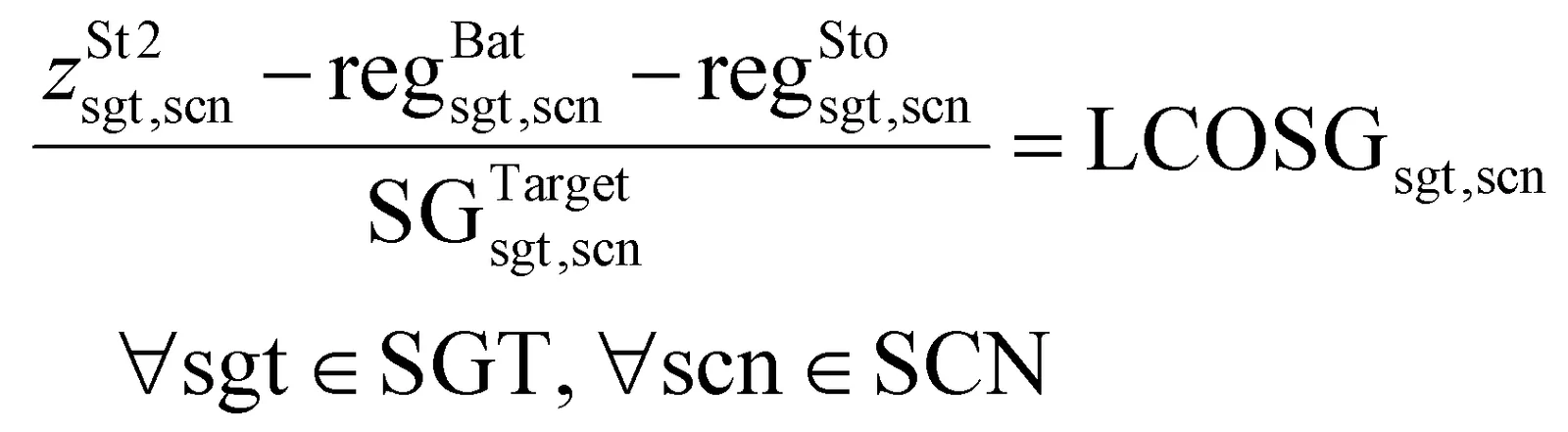




#### Renewable electricity generation

The investment costs of the renewable electricity plants were based on the 2025 cost estimates from the Danish Energy Agency.^45^ Solar PV, wind onshore, and wind offshore were considered in set RES. Investment costs were annualized using the capital recovery factor (CRF), calculated *via* plant lifetimes and real interest rates. Lifetime assumptions were drawn from the Danish Energy Agency,^45^ while the real interest rates from the different generation technologies were based on the Fraunhofer Institute.^46^ The cost of generation (cost^Gen^) was found by optimizing for the generation capacity (*c*^RES^_res,sgt,scn_) and the generation cost (cost^Gen^), using eqn (5). The CAPEX and OPEX data were stored in the parameters CAPEX^RES^_res_ and OPEX^RES^_res_.

CAPEX^RES^_res_ is defined as annualized cost per capacity (MEUR_2019_ per kW per year), while OPEX^RES^_res_ is defined as cost per generated amount of electricity (EUR_2019_ per MWh). GRD^Own^ represents the grid fees for getting the self-generated electricity delivered to the electrolyzer. CF^Elec^_{res,*t*}_ is the capacity factor of renewable electricity generation across all timesteps belonging to *T*.^40^

**eqGenCost**_**sgt,scn**_

5

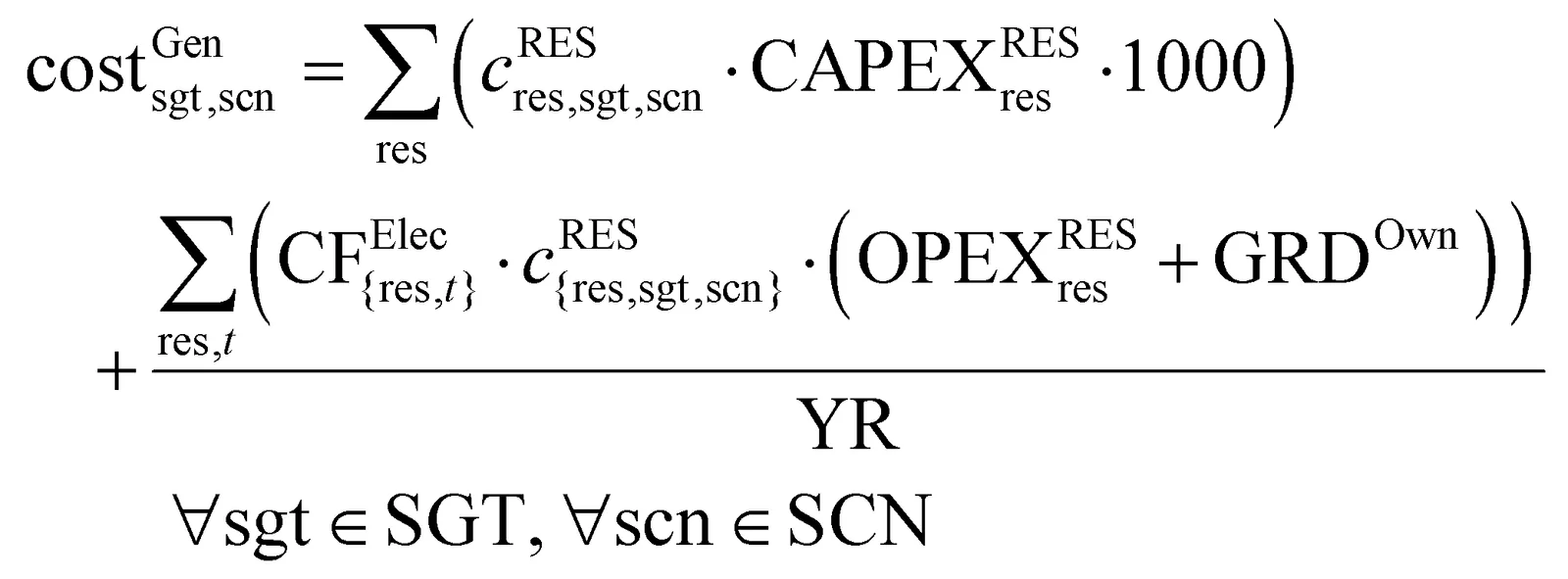




#### Battery

The lithium-ion battery cost data and lifetime were based on the 2025 cost estimates from the Danish Energy Agency.^47^ As a proxy, the real interest rate was based on the PV with battery interest rate of the Fraunhofer Institute.^46^ The total cost of operation and investment costs are collected in parameter BAT^Tot^ in EUR_2019_ per MW year. *c*^Bat^ is the selected capacity of the battery in MW.

**eqRegBatCost**_**sgt,scn**_

6

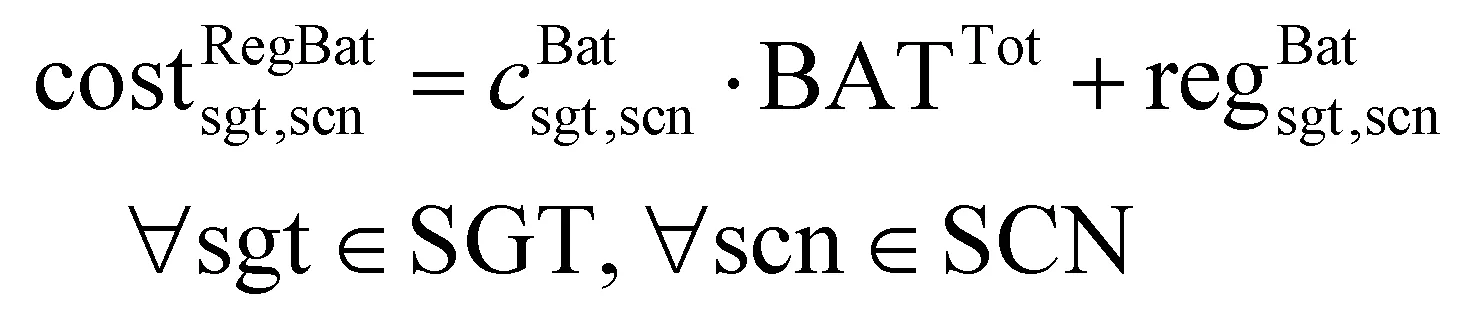




The battery in the first stage was described using a regularized LP relaxation approach based on the methodology of Han *et al.*^48^ Using this methodology from the literature, simultaneous charging and discharging are discouraged *via* cost regularizers in the objective function. In eqn (7), RG^C^ and RG^DC^ are small penalties for the charging (*p*^C^_*t*,sgt,scn_) and discharging (*p*^DC^_*t*,sgt,scn_) power, respectively. As a result, when the system is charging, discharging is penalized and therefore avoided, and *vice versa*. Furthermore, since RG^C^ is smaller than RG^DC^, the model slightly favors charging, ensuring that discharging occurs only when necessary. This approach allows for an accurate representation of battery charging and discharging behavior without introducing binary variables. Furthermore, it allows the solver to find a high-quality upper bound more easily, which improves the solving time. The lithium-ion batteries had an energy-to-power ratio of four, which is common for utility-scale storage systems.^49^ This ratio limits the maximum charging and discharging power of the battery.

**eqRegBat**_**sgt,scn**_

7

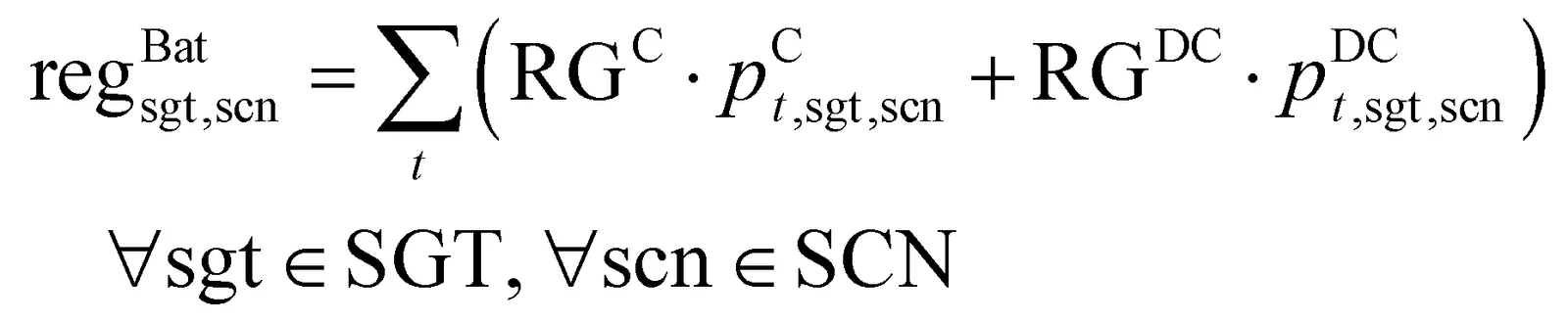




*e* ^SOC^_*t*,sgt,scn_ is the state of charge. The charging and discharging efficiencies were assumed to be 98% and 97%, respectively (ETA^C^ and ETA^DC^, Danish Energy Agency^47^). The energy balance of the battery is presented in eqn (8), where also a self-discharge value of 0.1% per day (SELF^DC^) was considered.^47^

**eqEnergyBal**_***t*,sgt,scn**_

8

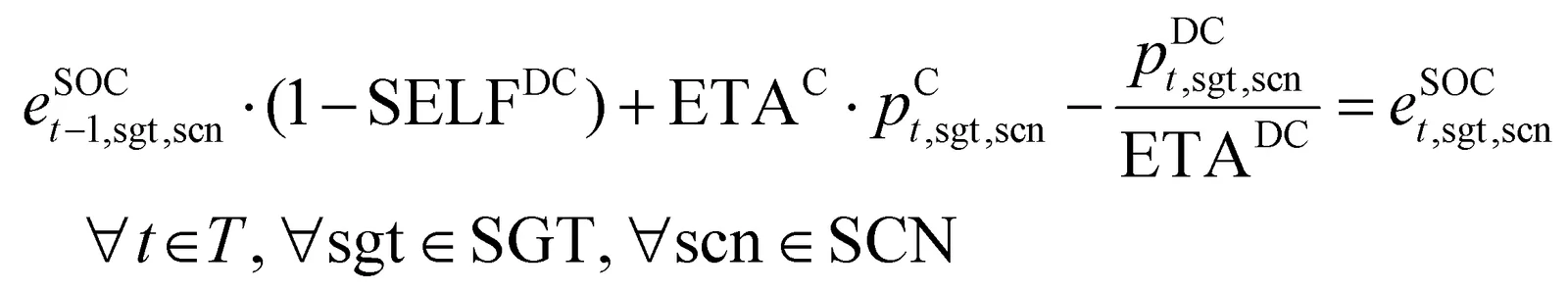




To improve longevity and maintain safe operation, the battery was operated between SOC^Min^ of 5% and SOC^Max^ of 95%.^50^ The state of charge is linked to the installed capacity of the battery *via*eqn (9) and (10).

**eqBat^MinC^_*t*,sgt,scn_**

9

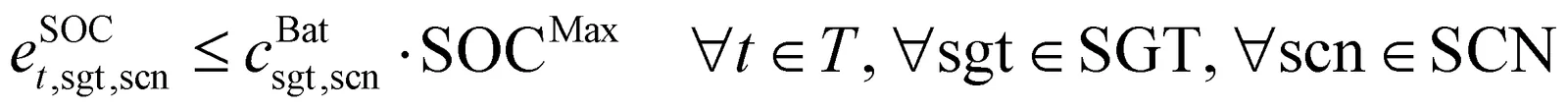




**eqBat^MaxC^_*t*,sgt,scn_**

10

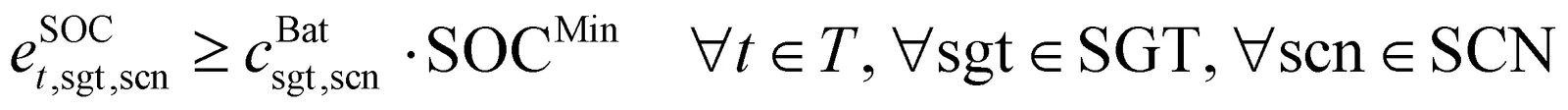




#### Syngas storage

The syngas storage costs were based on the work of Papadias and Ahluwalia.^35^ They investigated the bulk storage costs for pure hydrogen in an American context. Therefore, a translation to syngas and the European context was necessary. Corrections were made regarding the density difference between hydrogen and syngas. Also, correction factors based on material and labor differences were applied to account for contextual differences between the US and Europe.^51^ The same storage configuration was used as in the work of Papadias and Ahluwalia,^35^ where syngas was compressed to 100 bar and stored in 24-inch pipelines. These changes resulted in an annualized syngas storage cost (SG^Storage^, CAPEX and OPEX) of 11 800 EUR_2019_ per tonne syngas. The capacity of the underground pipeline storage system *c*^Sto^_sgt,scn_ was in tonne syngas. For all the changes made regarding the cost, see SI Syngas storage.

For underground pipeline storage, there was only a limited effect of economies of scale.^35^ Therefore, the pipeline storage system was assumed to scale up linearly as pipeline parts were added to reach larger volumes. The maximum capacity was unconstrained, and the pipeline storage system operated between 5% and 95% of its capacity.

The syngas leaving the electrolysis plant was conditioned to be directly usable in a Fischer–Tropsch synthesis plant at 220 °C and 30 bar pressure. To operate this storage system safely, the syngas was cooled down to 12 °C from 220 °C after the electrolysis plant. At elevated temperatures above 200 °C, hydrogen is known to cause hydrogen attack in carbon steel, which causes cracks to form.^52^

The syngas was cooled down to 12 °C to match the underground temperature using a water-cooled mechanical gas cooler with a COP of 3.^53^ Before FT synthesis, the syngas needed to be heated up again to 220 °C using an electric boiler at 99% efficiency.^54^ cost^StoHC^ includes the annualized investment cost for the syngas re-heating and cooling equipment. Eqn (11) calculates cost^RegSto^_sgt,scn_, which is the annualized cost of the syngas storage.

**eqRegStoCost**_**sgt,scn**_

11

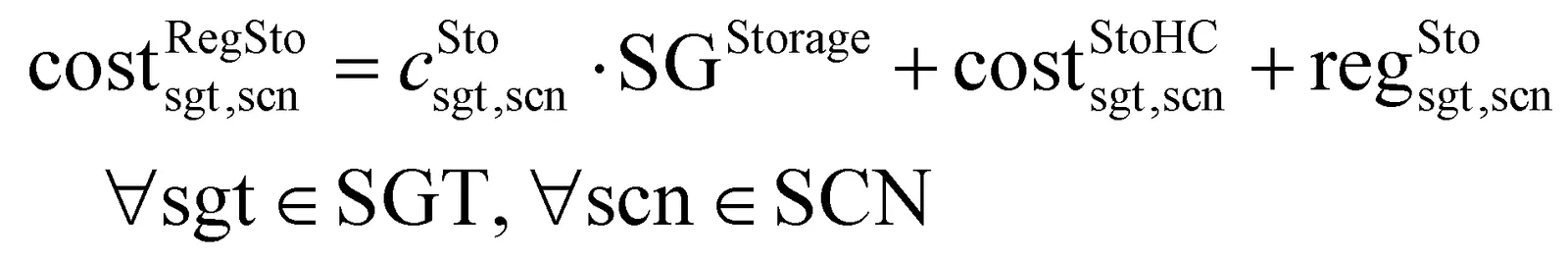




The electricity required for heating and cooling came from the own generation assets or the battery and was not dependent on grid electricity. The same regularized method was applied as for the battery to prevent loading and unloading at the same time. In this case, regularizers were applied to the heat required for loading (*q*^L^_*t*,sgt,scn_) or unloading (*q*^Un^_*t*,sgt,scn_) in kWh, see eqn (12).

**eqRegSto**_**sgt,scn**_

12

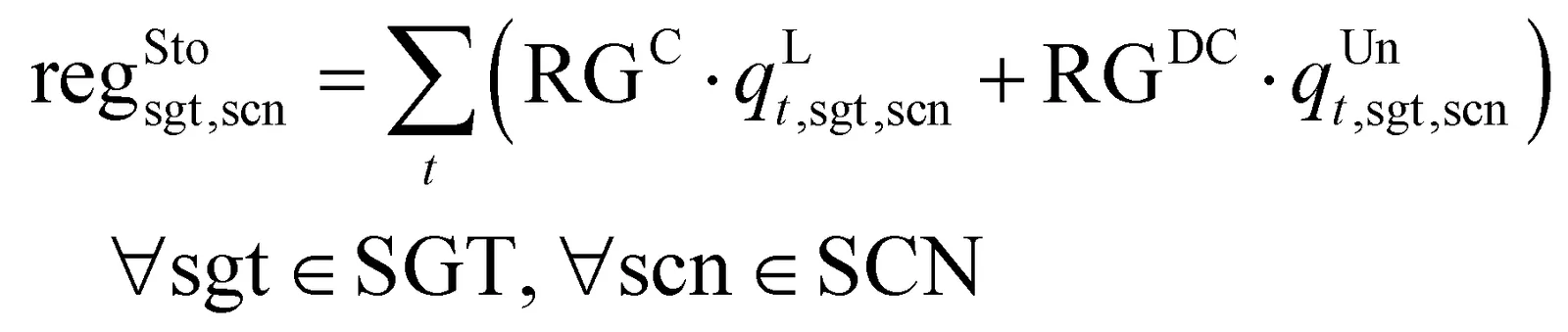




#### Electrolyzer

The type of electrolyzer used was a high-temperature solid oxide electrolyzer (SOE). CO_2_ and water are required inputs. However, the water requirement was not treated explicitly and was assumed to be available in the required quantities at the conversion site. Like in our previous work,^23^ we used a CAPEX of 2000 EUR_2019_ per kW, with a CRF of 0.128, which considered the replacement of stacks after 5 years of operation. The operations and maintenance costs were 4% of the CAPEX. With the electrolyzer size fixed, the investment cost was a fixed cost across different syngas targets.

**Fig. 4:**
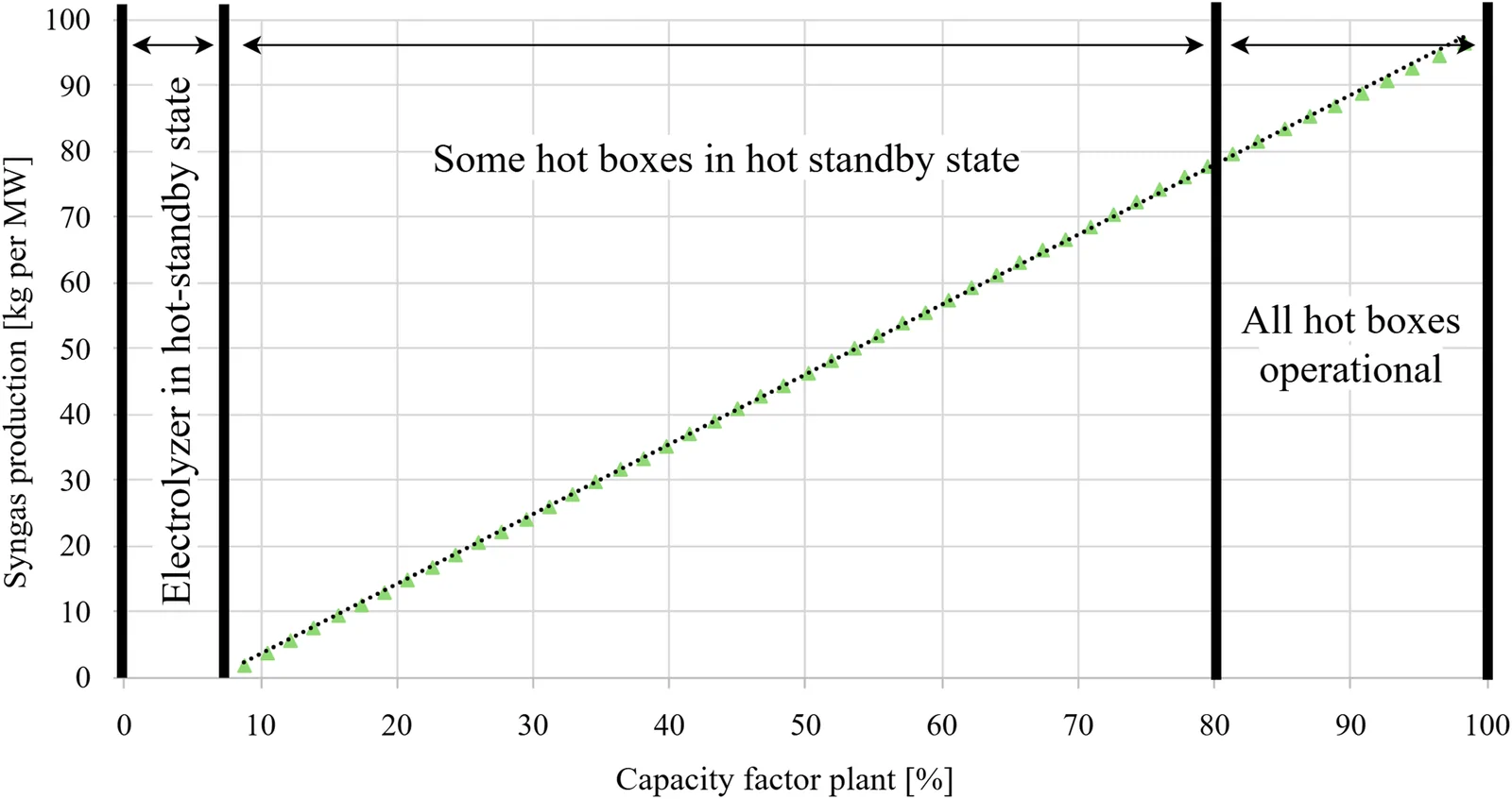
Linear syngas production regression line as a function of the capacity factor based on the optimization of the hot box electrolyzer submodel. The slope (*A*^Elyzr^) is 1.06 and the intercept (*B*^Elyzr^) is −7.22.

The syngas production of the electrolyzer within an hour was calculated using the trendline of Fig. 4. This regression line between the capacity factor of the electrolyzer and the production of syngas was found using a hot box operation electrolyzer submodel. This sub-model was developed for this work to be able to linearize the variable operation of an electrolysis plant taking into account the total number of hot-boxes. The 540 MW electrolyzer was assumed to consist of 360 hot boxes which could ramp up and down individually. The objective of this MILP submodel was to minimize the electricity consumption given a syngas production target by setting the hot-standby and operation state (between 32.5 and 100%) of the individual hot boxes. The hot box efficiency is modelled using a piecewise linear approximation: it is assumed to remain constant at its maximum efficiency of 92% for loads between 80% and 100%. For partial loads, ranging from 80% to 32.5%, the efficiency decreases linearly from 92% down to 77%. The full electrolyzer is completely in the hot standby mode below a 7.2% load. Between 7.2% and 80%, some hot boxes in the electrolyzer are in the hot-standby mode, while others operate at full or partial load. Above 80% all the hot boxes are operating at peak efficiency. The slope (*A*^Elyzr^) and intercept (*B*^Elyzr^) from the submodel regression line were needed to find the mass of syngas produced each hour (*m*^SG^_*t*,sgt,scn_), see eqn (13).

**eqProduction**_***t*,sgt,scn**_

13

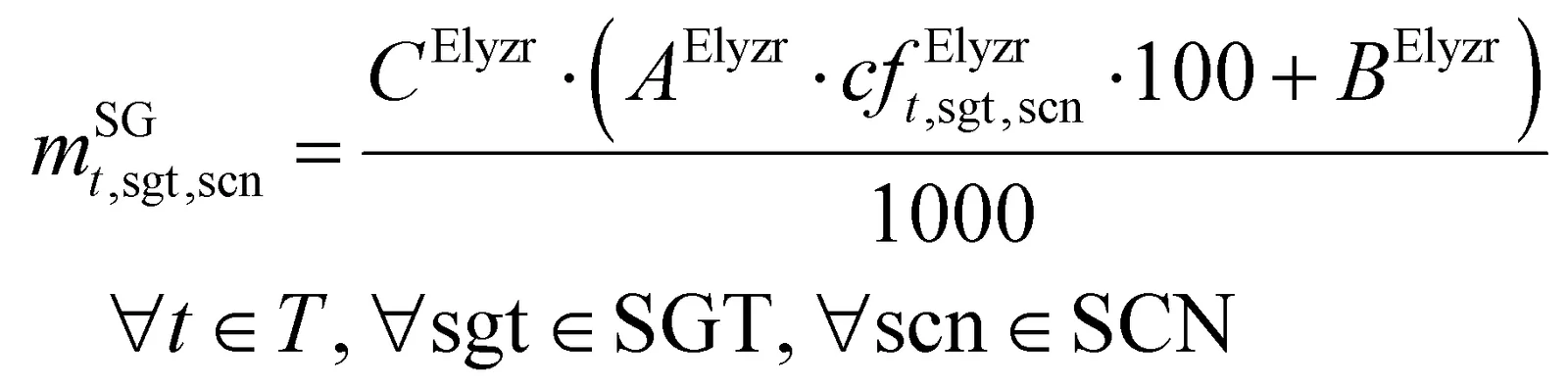




From the hot standby mode, the electrolyzer was able to ramp up within 3 minutes to full load.^31^ The loss in production during ramping time was not considered. The option to turn the system off completely was not included as thermal startup takes hours to days and may cause severe degradation.^30,55^

### 3.4 Background emissions of the SAF supply chain

This section specifies the inventory data and emission factors used to calculate the total carbon intensity (*G*^B^_scn_) of the e-SAF supply chain in accordance with RFNBO compliance standards.

The system is divided into foreground (co-electrolysis and renewable energy) and background stages. The background system encompasses: (i) CO_2_ capture and compression, (ii) CO_2_ transport, (iii) CO_2_ conditioning for use, (iv) FT synthesis, (v) fuel transport to the airport, and (vi) SAF end-use (Fig. 1). Crucially, the first four stages (i–iv) were assumed to be powered by grid mix electricity. Therefore, the GWP of the grid mix electricity directly impacts the amount of grid mix electricity that could be consumed in the electrolysis plant.

The assessment focuses exclusively on GWP and uses the specific accounting rules of the EU Delegated Acts (RED II/RED III) rather than the scope 1–2–3 framework. The GWP for RFNBO compliance is not a full life cycle assessment. For instance, emissions from the manufacturing of equipment and infrastructure are excluded (EC/2023/1184). The functional unit was 1 tonne of e-SAF used in a plane, with the emissions expressed in kg CO_2_-eq per tonne of e-SAF.

Table 2 summarizes the specific accounting parameters, data sources, and methodological choices. Specific adaptations from the source material can be found in the SI, Background emissions.

**Table 2:** RFNBO compliance accounting parameters and GHG emission factors

Parameter	Supply chain stage	Key assumptions	Source
*G* ^CC^_scn_	i. CO_2_ capture/compression	Bioethanol: compression and conditioning only. Pulp and paper: full capture and conditioning required	Nöhl *et al.*^56^, Emborg *et al.*^57^
*G* ^CT^_scn_	ii. CO_2_ transport	Emission factors (g CO_2_-eq per tonne km of CO_2_) applied to distances and flows defined in the supply chain design of Fig. 1	Nöhl *et al.*^56^
*G* ^CU^_scn_	iii. CO_2_ conditioning for use	Reduction of dense phase CO_2_ pressure (>80 bar) to gas phase pressure prior to CO_2_ co-electrolysis	Nöhl *et al.*^56^
*G* ^FT^_scn_	iv. FT synthesis	The process is assumed to be fully electrified. Impacts are mass-allocated between e-SAF and naphtha	Boilley *et al.*^25^
*G* ^ST^_scn_	v. e-SAF transport	Route: Rotterdam to Schiphol (150 km round trip). Mode: 5-long haul diesel truck (30 m^3^ cargo, 56.5 g CO_2_-eq per tonne km)	ACEA^58^
*G* ^EU^_scn_	vi. End use	CO_2_ from combustion is considered carbon neutral (biogenic origin). GWP accounts for non-CO_2_ effects (*e.g.*, water vapor and NO_*x*_), estimated at 1% of the total e-SAF SC GWP	Rojas-Michaga *et al.*^59^

The amount of grid mix electricity that can be consumed in an hour was based on the emission of a fossil comparator stemming from the EU's RFNBO regulation. According to the RFNBO regulation, a fossil benchmark (used interchangeably with the comparator) produces 94 g CO_2_-eq per MJ (EC/2023/1185). The emissions of fossil kerosene were estimated at 4089 kg CO_2_-eq per tonne kerosene, using a lower heating (net calorific) value (LHV) of 43.5 MJ kg^−1^.^60^ With an emission reduction threshold of 70% across the full supply chain, the e-SAF supply chain cannot emit more than 1227 kg CO_2_-eq per tonne SAF (*G*^T^). The different background emissions from the different supply chain stages were summed and subtracted from this threshold value. This resulted in a budget of the amount of CO_2_ emissions per kilogram of e-SAF that could be emitted while still meeting the RFNBO threshold (*G*^B^_scn_). The global warming potential budget was estimated using eqn (14).

**eqBudget**

14

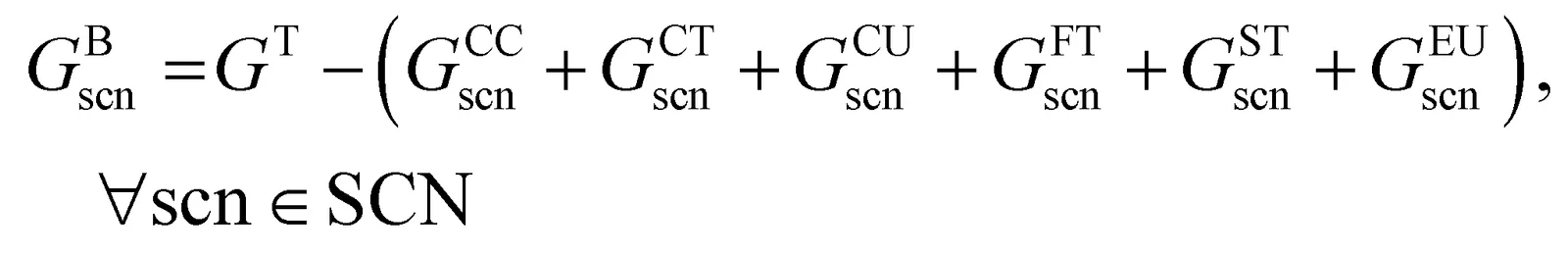




With eqn (15), the allowed grid mix percentage per hour is determined. GRID^GWP^_scn_ was set based on the different future scenarios and grid mix emissions following Table 1.

**eqGridBudget**

15

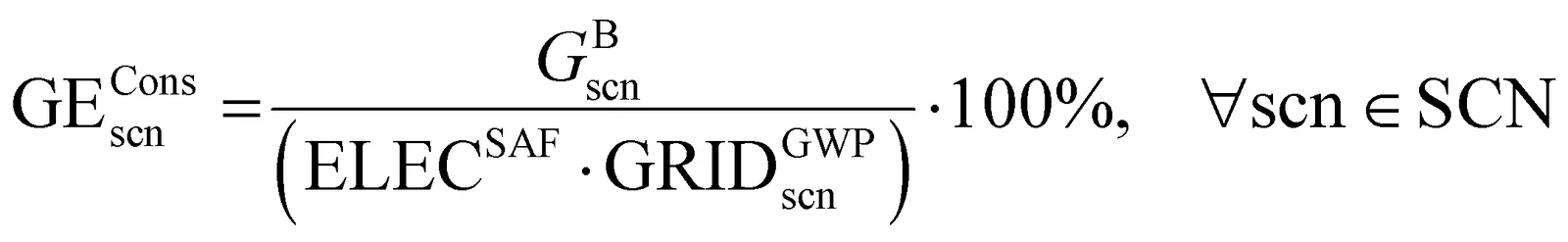




ELEC^SAF^ was calculated to be 29.0 MWh per tonne SAF based on the yield and efficiency factors of Noordende *et al.*^28^ and Boilley *et al.*^25^

## 4 Results

### 4.1 Background emissions and electricity grid mix budget

**Fig. 5:**
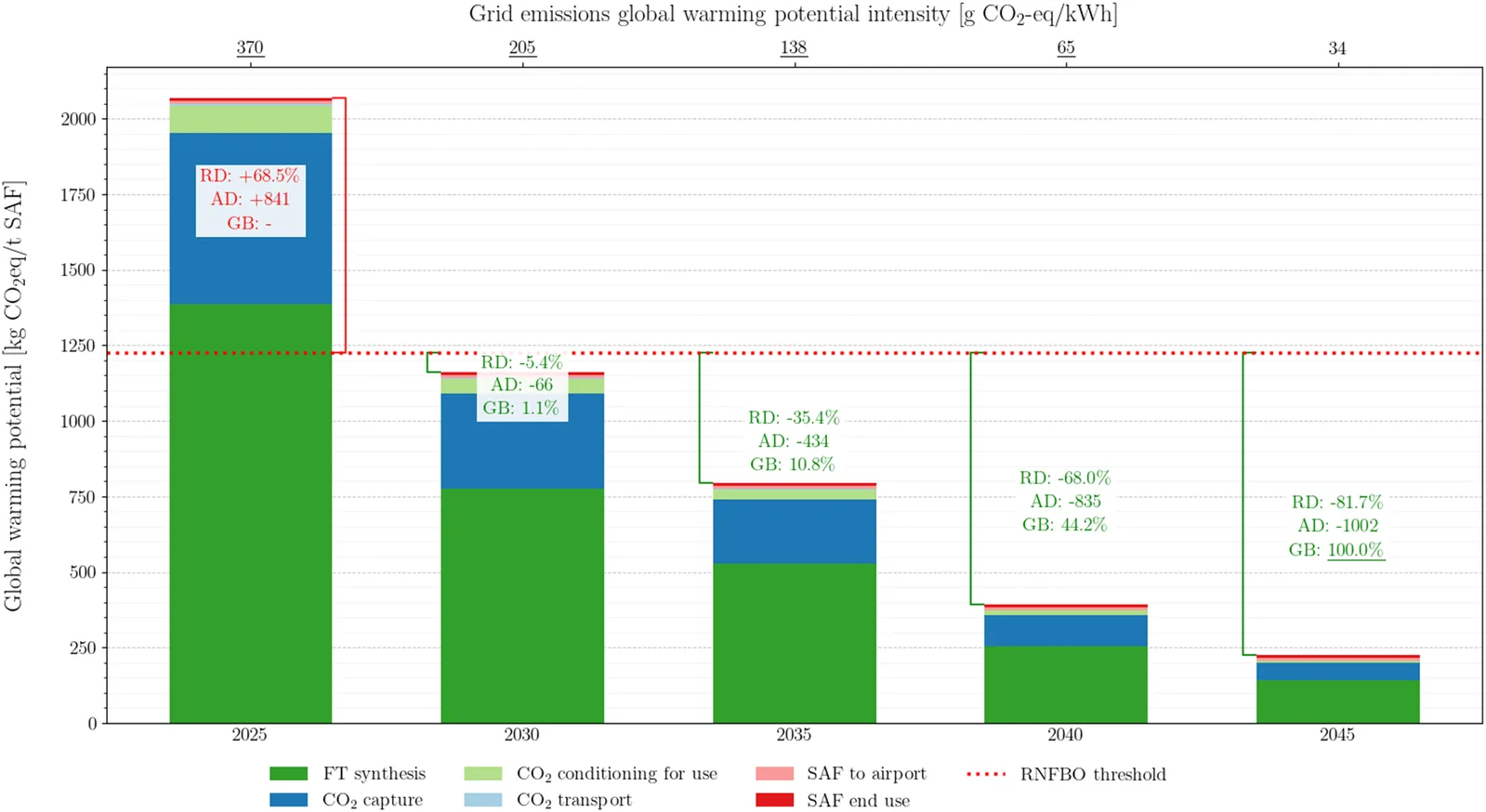
Global warming potential of the background stages. The underlined value is the limiting value across the future scenarios. RD stands for relative difference to the RFNBO threshold line and AD stands for absolute difference. GB stands for grid mix budget, the percentage of grid mix electricity that can be consumed per hour.

Fig. 5 displays the effect of the global warming potential of the background system elements for different grid emission intensities based on future scenarios. In 2025, when only considering the emissions from the background system, the RFNBO threshold is exceeded by almost 70%. With the emissions from the current Dutch electricity grid mix, it is not possible to produce RFNBO-compliant SAF. The FT section alone would emit enough to exceed the emission reduction threshold. Therefore, this scenario is not further considered in the rest of the results. The FT stage in the supply chain is responsible for 63–67% of the background emissions in all the cases, followed by the emissions from CO_2_ capture between 25 and 27% and CO_2_ conditioning with a contribution of around 4%. CO_2_ transport, e-SAF transport, and end use only have a minor contribution to the GWP. The contribution of CO_2_ transport is smaller when performing RFNBO emission accounting, since construction emissions are not included. For CO_2_ transport, these emissions are typically the main contributor to the global warming potential.^61^

From 2030 onwards, the background emissions are below the 1227 kg CO_2_-eq per tonne SAF threshold. This leaves some room for the electrolysis plant to consume 1.1% grid mix electricity per hour and still meet the RFNBO threshold. Since the RFNBO compliance needs to be proved per hour, exceeding this budget during any hour will still disqualify the compliance of the product during that hour. In scenario 2035-138, due to the grid mix emissions lowering, the grid mix budget increases to around 11%. When the grid mix emissions are below 65 g CO_2_-eq per kWh, additional restrictions on the consumption of the grid mix electricity of the electrolyzer are lifted. With the GWP threshold still being enforced, only 44.4% of grid mix electricity can be consumed. The grid mix emissions need to drop below 34.5 g CO_2_-eq per kWh to be able to consume grid mix electricity without any restrictions.

Table 3 presents the projected grid mix GWP scenarios when unlimited grid electricity could power the electrolyzer. Notably, this situation would result in a product with a significantly higher GWP than fossil-based alternatives in both the 2030-205 and 2035-138 scenarios. Consequently, strict constraints on grid usage are essential to ensure the process yields an environmental advantage over fossil fuels.

**Table 3:** Projected emissions when consuming exclusively grid mix electricity

	Unit	Fossil	2030	2035	2040	2045
Grid mix emissions	[g CO_2_-eq per kWh]	—	205	138	65	35
Absolute CO_2_ emissions	[kg CO_2_-eq per tonne SAF]	4089	7116	4802	2280	1227
Relative difference with the fossil comparator	[%]	0	+74	+17	−44	−70

### 4.2 Sizing of storage and renewable energy generation

**Fig. 6:**
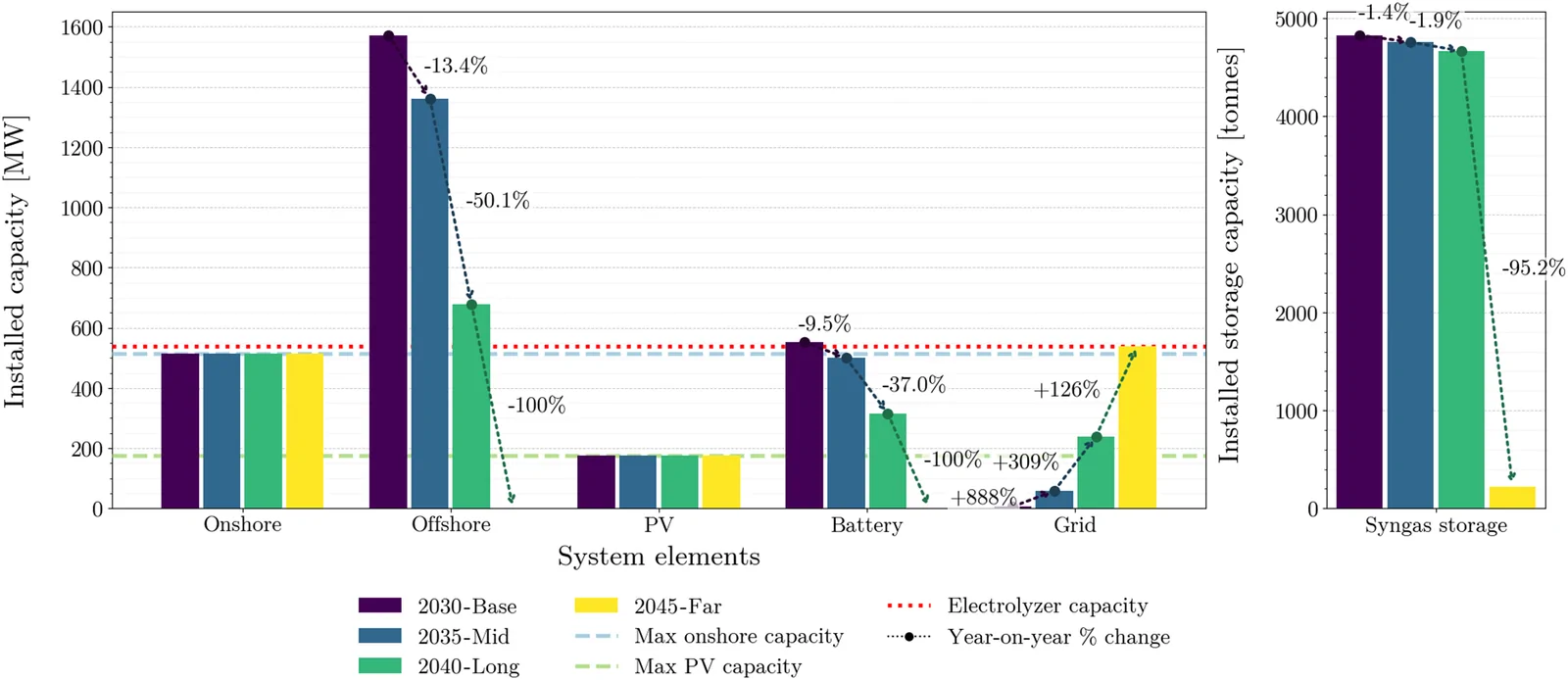
Sizing of the different optimization elements across the years at the lowest levelized cost point in the second optimization stage.

Fig. 6 shows the installed capacities of the different system elements across different scenarios. Onshore wind and PV hit the maximum constraint size in the model. This is independent of the grid mix penetration scenario. The size of offshore wind, however, decreases over the scenarios. Grid mix electricity replaces the need for additional offshore wind generation. In the 2030-205 scenario, offshore wind has a capacity which is 2.9 times higher than the electrolysis plant capacity. The battery has a capacity of 552 MW, which is in the same order of magnitude as the electrolysis plant itself (540 MW). With increasing amounts of grid mix electricity, progressively less offshore wind and battery capacity is needed. This rapid decrease in the battery and generation side is not mimicked in the installed syngas storage capacity from 2030 to 2040.

The FT plant requires a constant syngas flow throughout the year; however, energy generation is affected by seasonal variations. As a consequence, the storage of syngas needs to bridge these seasonal differences. The largest fraction of electricity comes from wind, with the highest average wind speeds in autumn and winter.^62^ Typically, storage is full at the end of winter and empty at the end of summer. This remains the case also in the 2040-65 scenario, where the largest fraction of electricity comes from wind. Only when all the electricity can be taken from the grid mix, there is no such seasonal effect and the syngas storage capacity decreases by more than 95%.

### 4.3 Levelized cost of syngas production

**Fig. 7:**
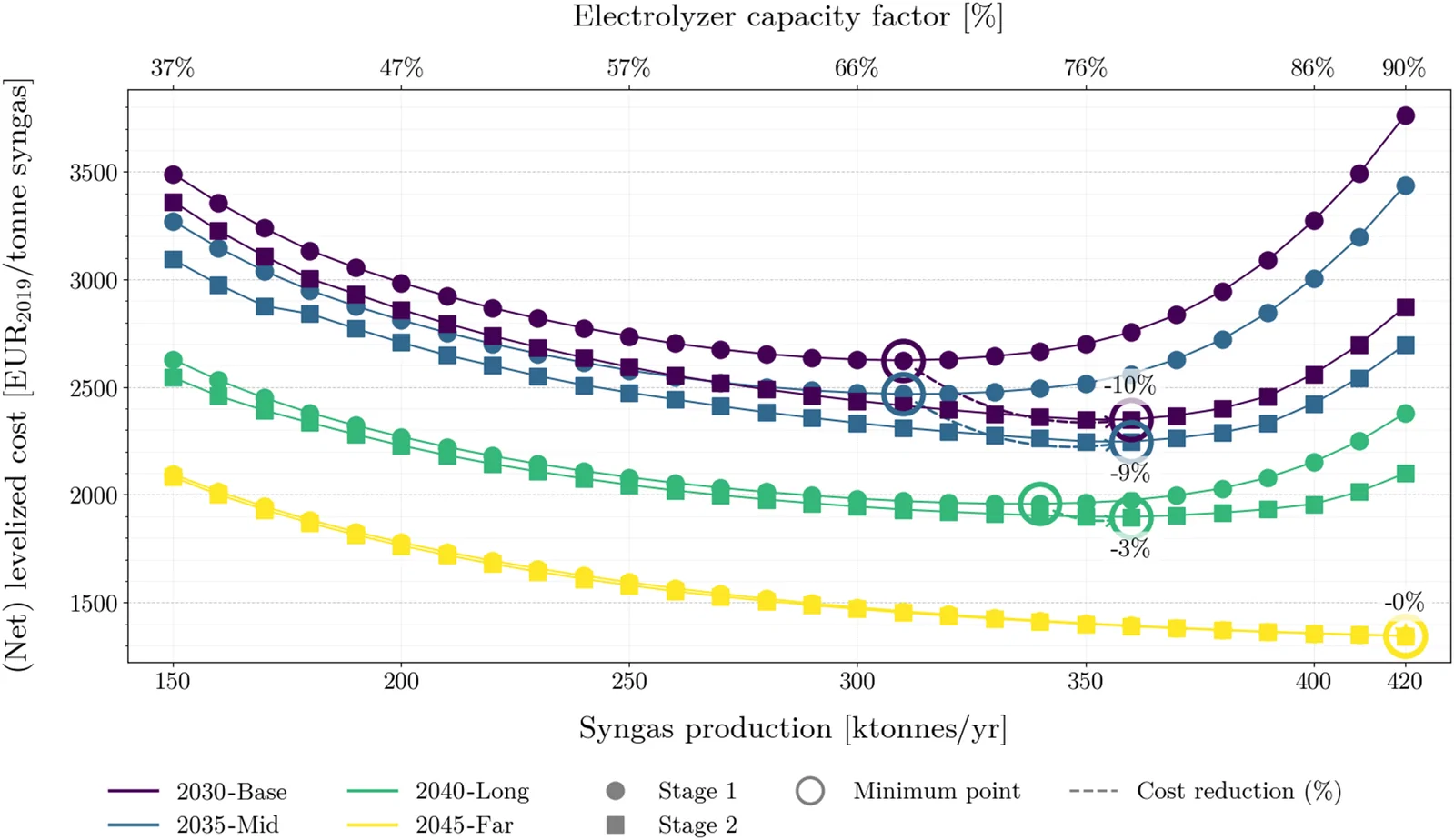
Levelized cost of syngas production across the different scenarios and optimization stages. The dotted arrows indicate a cost reduction between stage 1 and stage 2.

The different scenarios and optimization stages are plotted in Fig. 7. The dots mark the results in the first stage, where electricity cannot be sold back to the grid. The second stage is marked with squares. At this stage, selling electricity is permitted while operating the installed assets and battery to minimize the overall cost.

The different syngas targets per year affect the average capacity factor of the electrolysis plant, which is presented on the top axis of Fig. 7. This increase from 37% to over 90% occurs because higher annual targets force the plant to maximize its utilization, shifting from more intermittent operation (following renewables) to continuous operation (baseload). In the scenarios with restricted grid mix electricity consumption (2030–2040), there is an optimal levelized cost point, depicted by circles around the datapoints in Fig. 7. In the 2030-205 scenario, this minimum is at 310 ktonnes per year in the first stage. The levelized cost from stage 1 is 10.5% lower in the second stage with a syngas production quantity of 360 ktonnes per year.

When more grid mix electricity consumption is allowed, the levelized cost decreases from 2350 EUR_2019_ per tonne syngas in 2030 to 1344 EUR_2019_ per tonne syngas in 2045, see Table 4. The optimal levelized cost in the 2030-205 scenario is approximately 10 times higher than the cost of fossil-based syngas.^63^ When all the electricity can be bought from the grid, there is no local minimum anymore. The lowest levelized cost is realized when the plant operates at a continuous capacity factor above 90%.

**Table 4:** Development of optimal levelized costs in stage 2 across the different future scenarios: projected reductions relative to the 2030 baseline

			Reduction *versus* 2030 baseline	
Scenario	Scenario name	Net levelized cost	Absolute change	Relative change
—	—	(EUR_2019_ per tonne syngas)	(EUR_2019_ per tonne syngas)	(%)
2	2030-205	2350	Baseline	Baseline
3	2035-138	2247	−103	−4
4	2040-65	1897	−453	−19
5	2045-100%	1344	−1006	−43

### 4.4 Cost contribution of system elements

**Fig. 8:**
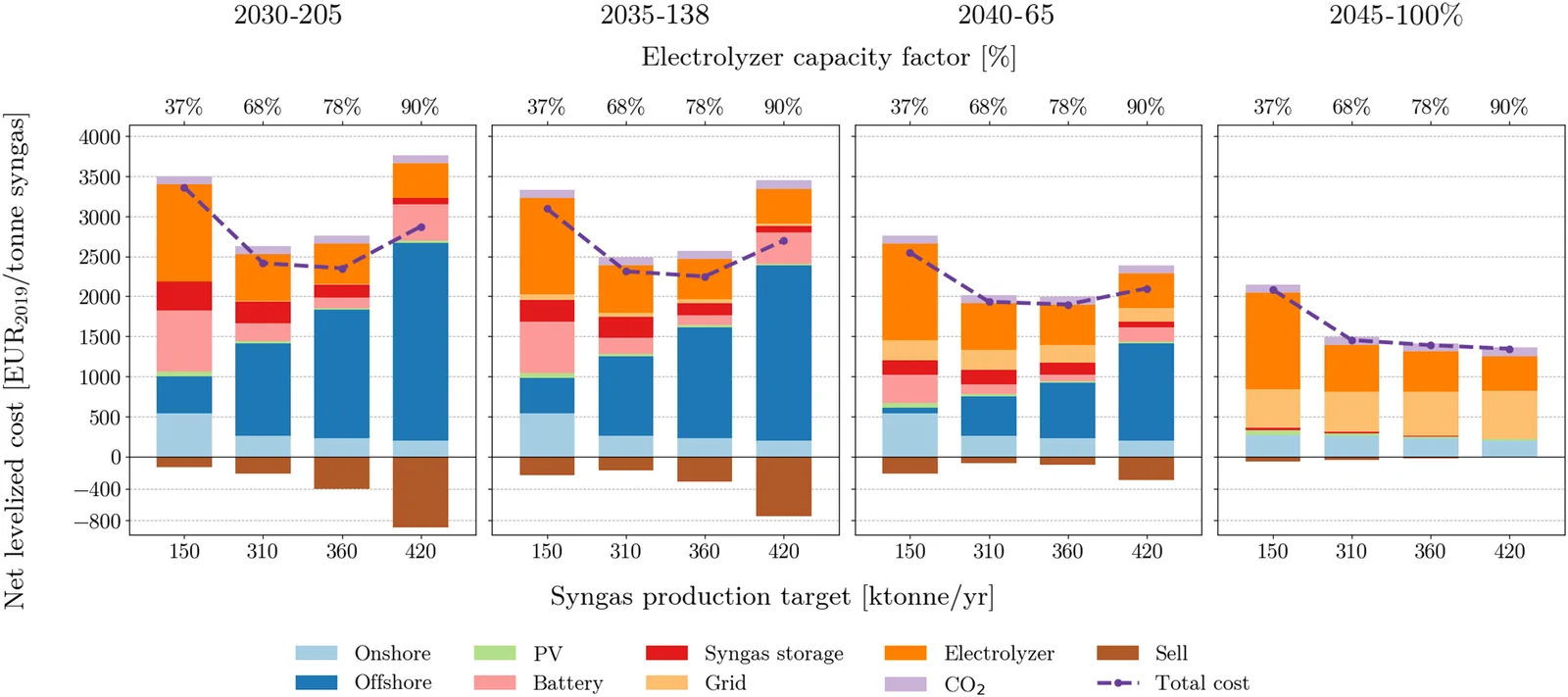
Stage 2 cost contribution of the different elements of the electrolysis plant and renewable energy system to the levelized cost of syngas production.

Fig. 8 gives an overview of the cost contribution of the electrolysis plant and renewable electricity system elements. It presents four different scenarios with four syngas production targets per scenario: minimum production, maximum production, and the optimal production levels for stage 1 and stage 2.

At low CFs, the costs are electrolyzer-dominated, whose contribution decreases when the grid mix electricity consumption increases. In 2030 at the lowest CF, storage and energy generation make up roughly the same share of the cost, at 30.1% and 32.1% respectively. The importance of the storage section (battery and syngas storage) in the cost decreases at higher capacity factors. Reaching the 90% capacity factor requires a battery capacity of 645 MW. This creates a major inefficiency, as a large part of this capacity is only needed during rare periods of very low renewable electricity generation. These additional investment costs cannot be compensated for by the additional revenue from selling electricity. With a large fraction of grid mix electricity that can be consumed, this effect decreases as battery storage and own generation become less necessary. However, before 2045, operating the electrolyzer at a reduced load through the hours of the year with too little generation remains a strategy that allows for lower production cost. In the current case study, the balance between generation, storage, and operation is achieved at 360 ktonne syngas per year with a capacity factor of 78% of the electrolyzer.

In 2035, a similar cost distribution among the system elements is visible. However, the extra grid mix electricity reduces the cost for offshore wind generation. A trend that continues in the 2040 scenario, completely removing the need for its own offshore wind generation in 2045. Having storage capacity is important when the system needs to provide its own renewable electricity. All cases up to and including 2040 use both batteries and syngas storage. Some generation from onshore wind and PV was still selected in the 2045-100% scenario. The grid mix will take over the function of guaranteeing electricity availability. Since there are still price fluctuations of the grid electricity and availability fluctuations of PV and wind generation, it is optimal to let the electrolyzer respond to these price and availability signals. Therefore, syngas storage is still needed; however, its size is greatly reduced, see Fig. 6.

## 5 Discussion

### 5.1 Grid connection

Establishing a grid connection is essential for optimizing the levelized cost. However, this requires ensuring that the consumed grid mix percentage in each hour remains under its threshold value. The rest of the grid electricity must meet the additionality, temporal correlation, and geographic correlation requirements of the RFNBO regulation. The electricity can be delivered from own generation assets or *via* power purchase agreements (PPAs). Introducing the option to sell electricity to the market increases the system's capacity factor and significantly reduces the levelized cost of syngas. Furthermore, a grid connection reduces the need for extensive storage assets and allows for more efficient utilization of battery capacity. In contrast, a CO_2_ electrolyzer configured in ‘island mode’ (operating *via* a direct line without grid access) cannot capitalize on these revenue streams and cost reductions. Consequently, this significant economic opportunity is missed. This places non-grid connected direct transmission line configurations at a distinct economic disadvantage. While not impossible to implement, these island-mode designs demonstrate significantly lower potential for future CO_2_ electrolysis scenarios compared to their grid-connected counterparts.

Furthermore, without grid access, the electrolyzer is constrained by the installed capacity of its dedicated power plant. Connecting to the grid opens the door to a broader portfolio of generators. This diversity reduces the variability of the combined power supply, which is a prerequisite for maintaining the continuous operation in FT synthesis. With the CO_2_ emissions in the grid mix dropping over time, the electrolysis grid connection becomes even more important, as the grid will supply a growing fraction of energy.

### 5.2 System sizing and optimization under uncertainty

The regulations regarding grid fees and generation feed-in tariffs are currently being developed.^64^ How exactly these rules will change the cost of electricity is currently unknown. Furthermore, a long-time frame was selected, which includes (i) a period of relative stability and gradual increases (2019–2021), (ii) the sharp price shock with high volatility linked to the gas crisis (2022–2023), and (iii) a phase of moderation and structural adjustment driven by renewables and policy measures (2023–2024). However, how the electricity prices will develop in the future is also highly uncertain,^65^ with uncertainty regarding the price level and price variations throughout the year.^66^

Addressing the price level first, a uniform increase or decrease in grid electricity prices primarily affects overall production costs without altering the optimal sizing. This insensitivity to a change in cost levels extends to the investment cost of the primary equipment. To illustrate this, in the current optimization problem, PV and onshore wind are utilized at their respective maximum capacities. Since these technologies are the cheapest sources in the merit order of electricity used in this work, they are exploited first. Unless the cost of offshore wind falls below the levelized cost of PV or onshore wind, which future projections do not anticipate,^45^ the cost of electricity production does not directly alter system sizing. The generation sizing will hold as long as the merit order and maximum capacity remain the same. Similarly, the electrolyzer has a fixed size and is not a design variable in the optimization. Its investment cost, therefore, does not affect system sizing, even though it remains a significant component in the levelized cost of syngas, which is further illustrated in Appendix A.

While absolute cost levels do not shift the sizing decisions, the aforementioned variations in prices and renewable generation profiles will impact this decision. The battery and syngas storage sizing is linked to moments with the lowest electricity production. During these periods, storage must supply minimum electricity to the electrolysis plant and syngas to the Fischer–Tropsch synthesis plant. Therefore, system design is affected by seasonal variations in PV and wind capacity factors. Changes in the variability and capacity factor profiles of renewable generation require the system design to adapt. Shifts in the timing of low generation, alongside time series price differences, alter the required sizing and behavior of the storage units and operation of the electrolysis plant. Another factor that impacts the syngas storage requirement is the lower bound of the FT synthesis. When this decreases due to greater operational flexibility, syngas storage requirements and their associated costs are expected to decrease as well.

Ultimately, the proposed design holds across different investment cost assumptions because the optimization is driven by temporal constraints. Upper limits on renewable generation, minimum FT synthesis bounds, and time-series data on capacity factors and electricity prices determine system sizing decisions. Because these temporal profiles and long-term market dynamics carry a lot of uncertainty, future work should integrate stochastic optimization or robust optimization alongside sensitivity analysis.^67–69^ This will provide greater certainty regarding the design decisions across a range of input variations. Moreover, such analysis could include a more elaborate scenario analysis where the investment costs of generation and battery storage develop with time. The investment costs for the energy generation, batteries, and electrolyzers are expected to keep decreasing.^45,47^ Therefore, lower investment costs impact the long-term syngas cost projections. The above-mentioned methodologies allow for dealing with the high level of uncertainty in the problem.

### 5.3 Positioning of co-electrolysis for RFNBO-compliant syngas production

To evaluate the potential advantages and limitations of the proposed high-temperature co-electrolysis (SOEC) system, it must be positioned within the broader technological landscape of renewable syngas generation. The scope of this comparison is strictly limited to pathways that can generate syngas for RFNBO-compliant e-SAF using renewable electricity and biogenic CO_2_. Consequently, conventional pathways of producing SAF that do not rely on syngas intermediates are excluded (*e.g.*, lipid-based pathways or alcohol-to-jet (ATJ)). Similarly, while biomass gasification is a highly mature syngas production route,^70^ its reliance on biomass primarily yields bio-SAF rather than RFNBO-compliant e-SAF. Therefore, it is not further considered in the present analysis.

From a process design perspective, the feasible plant configurations for RFNBO-compliant syngas production include: (i) Reverse Water–Gas Shift (RWGS) coupled with green hydrogen production, (ii) (high and low temperature) CO_2_ electrolysis to CO followed by downstream green hydrogen blending to the right syngas ratio, and (iii) direct (high temperature) co-electrolysis of water and CO_2_. As established in recent techno-economic analysis and technology assessments,^71,72^ these configurations currently represent the options with the highest TRL for scalable e-SAF production.

Low-temperature water electrolysis is commercially mature (PEM or alkaline TRL 8–9 (ref. 73)), which, when combined with RWGS (TRL 6–7 (ref. 72)), is an alternative configuration for near-term syngas production for e-SAF production. A key advantage of this process route is the decoupling of hydrogen and CO_2_ conversion steps, which provides operational flexibility and mitigates technical risks.^74^ However, the endothermic nature of RWGS (typically operated at 550–950 °C^75^) requires process heat integration to minimize operational expenditures (OPEX).

Alternatively, the dedicated electrochemical reduction of CO_2_ to CO offers different process benefits. Low-temperature CO_2_ electrolysis (TRL 5–6 (ref. 76)) theoretically provides optimal flexibility for dynamic, start-stop operation with intermittent renewable electricity. Nevertheless, overpotentials and electrolyzer degradation lead to poor energy efficiency, which increases the production costs of syngas.^77^ Operating dedicated CO_2_ electrolysis at high temperatures (solid oxide technology) can mitigate kinetic limitations and improve CO_2_ conversion efficiencies.^78^ Moreover, this process step has the highest TRL (8 (ref. 72)) of the considered routes that can produce syngas. However, a drawback of separate CO_2_ reduction is the need for additional independent water electrolysis and downstream gas-mixing infrastructure to ensure the correct syngas ratio for the FT plant.

Within this techno-economic landscape, the high-temperature co-electrolysis (TRL 5–6 (ref. 73)) investigated in this work occupies a uniquely integrated position. By simultaneously reducing water and CO_2_ under near-thermo-neutral conditions, SOEC achieves unmatched thermodynamic efficiency and directly yields the syngas intermediate in a single unit operation,^79^ bypassing the need for separate gas blending infrastructure.

Future work should consider more elaborate sensitivity analysis, stochastic optimization, or robust optimization to have more certainty regarding the design decisions over a range of input variations and costs.^67–69^ Moreover, such analysis could include a more elaborate scenario analysis where the investment costs of generation and battery storage also develop with time. The investment costs for the energy generation, batteries, and electrolyzers are expected to keep decreasing.^45,47^ Therefore, the lower investment costs impact the long-term syngas cost projections and could affect the optimal configuration surrounding the electrolysis plant. The above-mentioned methodologies allow for dealing with the high level of uncertainty in the problem.

### 5.4 Scaling

Economies of scale in different system elements were not considered. The decision was made to run a simplified linear model, which has better solver performance over a longer time horizon. Within the system boundary, only technologies that are highly modular were considered. Furthermore, the system designed in this work operates at a scale where the different elements need to be numbered up in order to reach larger scales. This results in a smaller effect of economies of scale compared to a conventional chemical process.^80^ When the FT synthesis with the downstream processing is part of the system boundary, traditional economies of scale need to be considered.^81^ The current amount of syngas produced for FT synthesis is on the small side of FT plants currently operational.^27^ In other words, further scaling down the employed modular system will negatively impact the economies of scale in the FT plant, having the potential to reduce the economic competitiveness of the system. Whether the loss of economies of scale in the FT plant is effectively compensated for by the modular scaling of the electrolyzer, battery storage, and renewable electricity generation is detailed in our extended work.^82^

### 5.5 Syngas bulk pipeline storage

In the optimal proposed design, large amounts of syngas need to be stored in the multi-ktonne range.

The baseline underground pipeline storage system requires substantial land area. For 1 ktonne of syngas, approximately 32 hectares of land are required. This value is based on the same area factors corrected for syngas as used by Papadias and Ahluwalia.^35^

In the port of Rotterdam area, space is scarce; therefore, a more space-efficient storage option would be desired in this case. Alternatively, pipelines could be stacked in a Tube Storage Tank Module (TSTM)^83^ to significantly minimize the space footprint, and the lack of widespread implementation makes accurate cost estimation difficult.

Consequently, pressurized vessels present an alternative engineering solution, particularly given that syngas is stored in the gas phase and must meet the 30 bar limit required by the FT-plant.^84^ In this pressure regime, spherical tanks (Horton spheres) are a feasible option to temporarily store syngas. Due to manufacturing constraints, there is a maximum limit to the feasible size of a single sphere. Pressurized spherical tanks are reported in industry^85,86^ between 1000 m^3^ and 5000 m^3^, which correspond to 13 and 65 tonnes of syngas (H_2_/CO ratio: 2) at 30 bar, respectively. The massive storage volumes required for continuous plant operations cannot be met by increasing the unit vessel size. Instead, the system must rely on the installation of multiple parallel spherical tanks. Spherical tanks also use height rather than ground area. Consequently, spheres therefore occupy a relatively small footprint per unit of stored capacity compared to single-layer piping.

Future work should quantify the cost implications of using a more space-efficient storage method that fits the Rotterdam spatial context by comparing the costs of spherical tank storage *versus* pipeline-based storage. Since the cost contribution of storage to the levelized cost of syngas is small in the optimal designs (<6%), it is not expected to impact the outcomes of this work. However, it will impact the deployment potential of the proposed designs. Alternatively, syngas production could move to locations where there is access to large-scale geological syngas storage (*e.g.*, salt or lined rock caverns).

### 5.6 Application of the model to other case contexts

This case study is tailored to the Dutch context and a specific e-SAF background supply chain in the Netherlands. This context directly influences the scenario timelines and its associated grid mix GWP. However, since the RFNBO regulation applies EU-wide, these findings have broader relevance. Countries such as Finland and Sweden already exhibit low grid carbon intensities^19^ that are projected for the Netherlands in the future. This makes these countries immediate candidates for full grid-connected operations. Therefore, even though the case inputs are specific to the Netherlands, the insights regarding sizing and grid-supported design philosophy are transferable to other contexts. Nevertheless, for the Netherlands specifically, the most cost-effective production route utilizing full grid support while complying with the RFNBO regulations is not expected to be feasible within the current decade.

By changing the input capacity factors, upper limits of generation and day-ahead electricity cost, the methodology can be applied throughout Europe. Using the presented model, in a future work, the most feasible locations and bottlenecks in different areas can be identified. In particular, areas with a high solar and onshore wind potential are interesting or where the grid mix is ready for the full grid mix operation. In such an analysis, the issue of where the biogenic CO_2_ comes from must also be considered. This has less impact on the final costs but can ultimately determine whether the system can be developed on a relevant FT scale.

## 6 Conclusions

The current work optimized the sizing and operation of a 540 MW CO_2_ electrolysis plant at different grid mix global warming potential intensities. The goal was to explore whether it was possible to produce RFNBO-compliant syngas with applications in e-SAF by using a combination of grid mix and renewable electricity from PV, onshore wind, and offshore wind.

When the electrolysis plant has the option to sell electricity back to the grid, the optimal number of operating hours increases from 68% to 78% in a 2030-205 scenario. A capacity factor of 78% is optimal for the scenarios where the consumption of grid mix electricity is restricted (>34.5 g CO_2_-eq per kWh). In a scenario where the plant can rely fully on grid mix electricity, the lowest production costs (EUR_2019_ per tonne syngas) occur at capacity factors above 90%. In this case, there is no need to invest in battery storage or offshore wind generation, as the grid supplies electricity when needed.

For the 2030–2040 scenarios, where the grid emissions range from 205 to 65 g CO_2_-eq per kWh, respectively, renewable energy generation and storage together account for 60–80% of the production costs. The cost of the electrolysis plant makes up approximately 10% of the production costs. The costs of grid mix electricity and CO_2_ supply are responsible for the remainder. In 2030, the capacity of offshore wind is 2.9 times larger than that of the electrolysis plant, while the battery has a comparable size to the electrolyzer. Onshore wind and PV solar were constrained in the model to reflect the Dutch context, but together they emerged as the preferred technologies for energy generation. To interface an intermittent electricity-operated electrolysis plant with a base-load Fischer–Tropsch synthesis plant, seasonal syngas storage is required. This leads to the need for multi-kilotonne (<5 kilotonne syngas) storage capacity. The size of syngas storage decreases by more than 95% when unrestricted grid electricity is available.

In 2025, the Dutch grid mix was not ready for industrial production of SAF *via* CO_2_ co-electrolysis. From 2030 onwards, the grid mix should be able to accommodate SAF production, as the background emissions of the SAF supply chain are reduced. This enables compliance with the RFNBO regulations. There is a significant difference between the syngas production cost in a 2030-205 scenario (2350 EUR_2019_ per tonne syngas) compared to a fully grid scenario in 2045-100% (1344 EUR_2019_ per tonne syngas). From a cost perspective, operating the electrolysis plant solely on grid electricity appears to be attractive; however, in the Dutch context, this is unjustifiable from an emissions perspective. Nevertheless, this grid operation mode may still be applied in other countries with lower grid intensities.

Future work can apply the presented model to identify the most feasible locations and bottlenecks across regions. Incorporating sensitivity, stochastic, or robustness analyses would further strengthen confidence in sizing decisions under varying inputs and investment costs.

## Author contributions

Thijmen Wiltink: writing – original draft, methodology, investigation, formal analysis, data curation, conceptualization, and visualization. Andrea Ramírez: writing – review & editing, supervision, methodology, and conceptualization. Mar Pérez-Fortes: writing – review & editing, supervision, project administration, methodology, funding acquisition, and conceptualization.

## Conflicts of interest

The authors declare that they have no known competing financial interests or personal relationships that could have appeared to influence the work reported in this paper.

## Abbreviations

CAPEX: Capital expenditures
CRF: Capital recovery factor
e-SAF: Electro-fuels for sustainable aviation fuel; a sub-category of SAF under the EU regulation
GWP: Global warming potential
LHV: Lower heating (net calorific) value
OPEX: Operational expenditures
PPAs: Power purchase agreements
SAF: Sustainable aviation fuels
SOE: Solid oxide electrolyzer
TSTM: Tube storage tank module

## Supplementary Material

GC-028-D6GC04304F-s001

## Figures and Tables

**Fig. 9:**
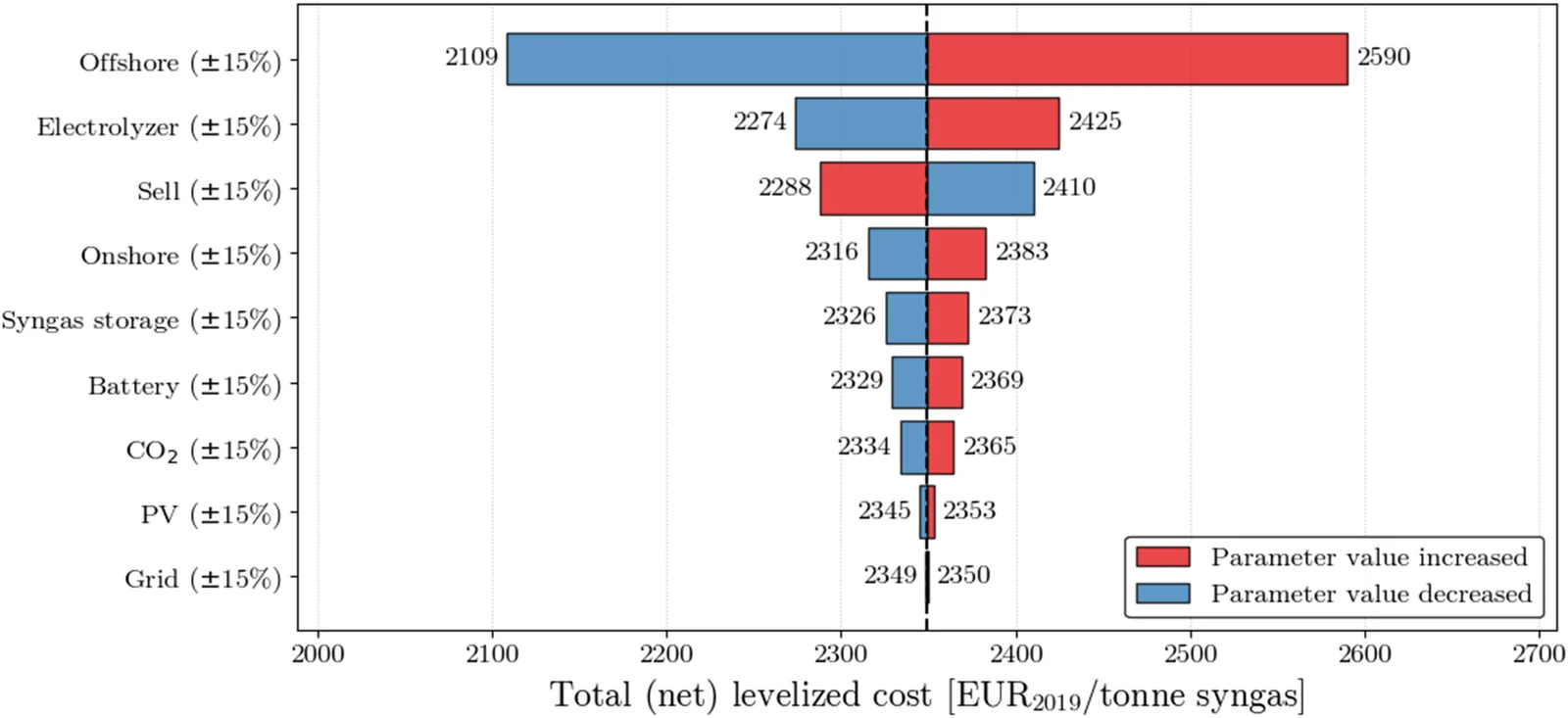
Tornado plot with a 15% increase and a decrease of cost across the different elements in the system for the 2030-205 scenario at a production of 360 ktonne syngas per year. The baseline costs are 2350 EUR_2019_ per tonne syngas. The system elements that are impacted most by the cost change are at the top of the figure.

**Fig. 10:**
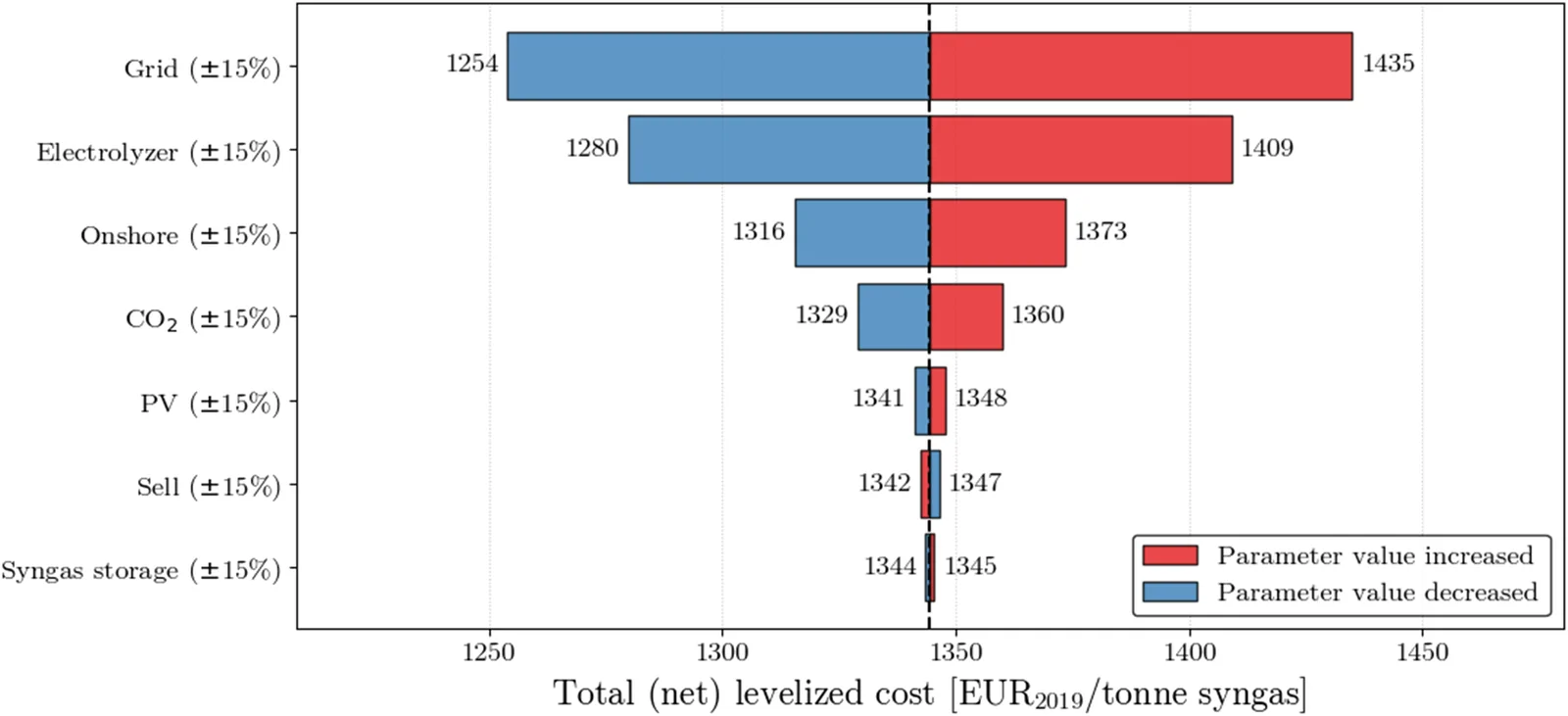
Tornado plot with a 15% increase and a decrease of cost across the different elements in the system for the 2045-100% scenario at a production of 420 ktonne syngas per year. The baseline costs are 1344 EUR_2019_ per tonne syngas. The system elements that are impacted most by the cost change are at the top of the figure.

## Data Availability

Supplementary information (SI) GIS-based supply chain data, runnable GAMS optimization models with input files, the mathematical formulation, renewable energy profiles, electricity prices, investment costs, and supporting datasets are available at https://doi.org/10.5281/zenodo.21813233. Supplementary information is available. See DOI: https://doi.org/10.1039/d6gc04304f.

## Appendices

### Appendix: A – Tornado plots of the levelized cost of syngas production

Fig. 9 and 10 present the effect of an increase and decrease of 15% of the costs across different elements in the system. For these figures, the system was not re-optimized with the changed cost input data; instead, the change in cost was applied to the optimally sized design.
