# Supplementary material for: Optimal sizing of renewable generation and storage for grid-connected CO2 electrolysis: a Dutch case study on syngas production for e-SAF
Source: Green Chem. 2026 Aug 19;28(35):14278–96. doi: 10.1039/d6gc04304f (PMC13487491; doi:10.1039/d6gc04304f)
Supplement: Supplementary file 1 [file GC-028-D6GC04304F-s001.pdf]

Supplementary Information (SI) for Green Chemistry.
This journal is © The Royal Society of Chemistry 2026

Symbols

Sets

Name	Domains	Description
T	*	Number of hours considered in the optimization
R	*	Selection of the NUTS-2 Region, NL for Netherlands wide
RES	*	Selection of the RES technology
$SCEN$	*	Set with cost data for different scenarios linked to years. 2025 is the base scenario
BAT	*	Selection of the battery storage technology

Parameters

Name	Domains	Description
YR		The number of years that the optimization considers
$CF^{\text{Elec}}_{res,t,r}$	RES, T, R	Capacity factor of the energy generation assets, combining onshore, offshore, PV
$OPEX^{\text{RES}}_{res,scen}$	$RES, SCEN$	OPEX - Operations and Maintainance cost of generation [EUR2019/MWh] based on Danish Energy Agency (2025)
RES^{Fixed}_{res}	RES	Annualized electricity generation CAPEX [EUR2018/MW*yr]
GRD^{Own}		Grid fees charged on your own produced electricity (10% lower than buy) [EUR/MWh]
GRD^{Buy}		Consuming external electricity via the grid, grid costs (higher than consuming own electricity) [EUR/MWh] based on Adriaansens & Jetten (2021)
BAT^{CAPEX}_{bat}	BAT	Annualized battery CAPEX [EUR2019/yrMW]
ETA^{C}		Charging eifficiency of storage technologies [-] based on Danish Energy Agency (2025)
ETA^{DC}		Discharging efficiency of storage technolgoies [-] based on Danish Energy Agency (2025)
C^{Max}_{bat}	BAT	C-rate of the different batteries [-]
P^{Max}		Maximum power that can be charged/discharged from the battery [MW]
$SELF^{DC}$		Amount of selfdischarge %/h based on Danish Energy Agency (2025)
RG^{C}		Regularizer for charging based on the methodology of Han et al. (2024)

RG^{DC}		Regularizer for discharging based on the methodology of Han et al. (2024)
SOC^{Min}		Minimum SOC fraction based on Rangelova & Jones (2025)
SOC^{Max}		Minimum SOC fraction based on Rangelova & Jones (2025)
C^{Elyzr}		Capacity of the electrolysis plant [MW]
CF^{Lo}		Lower capacity bound on the capacity factor, where there is no syngas production [-]
A^{Elyzr}		Slope of the syngas production curve as a function of the capacity factor [cf/(kg*MW)] based on post processing of sub-model
B^{Elyzr}		Intercept of the syngas production curve as a function of the capacity factor [kg/MW] based on post processing of sub-model
$ELEC_t^{\text{Price}}$	*	Market selling price of electricity [EUR/MWh] based on ENTSO-E (2025)
GE^{Cons}		Percentage per hour that can be consumed as grid electricity [%]
ST^{MaxL}		Maximum loading and unloading capacity [tonne/h]
ST^{Max}		Maximum storage mass of the tank [tonnes]
CPM^{SG}		Heat capacity syngas (2 H2: 1 CO) [J/(kg*K)]
D^t		Temperature difference input 220 °C output 12 °C [K]
CRF^{Sto}		CRF of the storage 30 year 8% [-]
$CAPEX^{\text{Heater}}$		The CAPEX in EUR/kW for the electric gas heater based on data from Agora Energiewende (2022) [EUR2019/kW]
$CAPEX^{\text{Cooler}}$		The CAPEX in EUR/kW for the electric gas cooler based on data from Fraunhofer IEG (2024) [EUR2019/kW]
COP^{Cooler}		The COP for cooling down the gas stream to 12 °C using a compression cooler from Fraunhofer IEG (2024) [-]
COP^{Heater}		The COP for (pre-)heating the gas stream to 220 °C using an electric heater from Agora Energiewende (2022) [-]
SG^{Storage}		Total storage cost factor based on Bulk storage of hydrogen [EUR2019/t] based on Papadias & Ahluwalia (2021)
SG^{Prod}		Scenario input, scalar regarding the annual syngas supplied to a SAF plant [ktonne/yr]

Variables

Name	Domains	Description
c^{RES}_{res}	*	Power capacity of the renewable energy generation [MW]
$gen^{\mathrm{In}}_{res,t}$	*, *	Energy generated by the renewable energy generation [MWh]
$cost^{\mathrm{Gen}}$		Cost for generating electricity (annualized, CAPEX and OPEX) [EUR2019/yr]
e^{Elyzr}_{t}	*	Energy that is directly used in the electrolysis plant [MWh]
e^{Curt}_{t}	*	Energy that needs to be curtailed or that is not used at all [MWh]
e^{Bat}_{t}	*	Energy that is stored in the battery [MWh]
e^{H}_{t}	*	Energy that needs to be used for heating the stored syngas to 220 °C [MWh]
e^{C}_{t}	*	Energy that needs to be used for cooling syngas to 12 °C for storage [MWh]
$u_{bat,t}$	*BAT, T*	Indicator for charging mode 0= discharging, 1= charging (u in [0,1])
c^{Bat}_{bat}	*	Overall yearly installed capacity of the battery [MW]
$p^{\mathrm{C}}_{bat,t}$	*BAT, T*	Charging power [MW]
$p^{\mathrm{DC}}_{bat,t}$	*BAT, T*	Discharging power [MW]
$e^{\mathrm{SOC}}_{bat,t}$	*BAT, T*	State of charge in the battery/ Available energy [MWh]
$e^{\mathrm{Net}}_{bat,t}$	*BAT, T*	Net charged/discharged energy from the battery [MWh]
reg^{Bat}_{bat}	*BAT*	Regulizer variable for the storage cost, where charging and discharging simultaneously are discouraged
$cost^{RegBat}$		Total regulated cost of the battery
$cost^{\mathrm{Bat}}_{bat}$	*BAT*	The actual battery storage costs without regulizers
cf^{Elyzr}_{t}	*	Capacity factor of the electrolysis plant during an hour [-]
m^{SG}_{t}	*	Variable for the syngas that is produced in each hour [tonne]
e^{Grid}_{t}	*	Electricity used from the grid [MWh]
e^{Input}_{t}	*	Electricity input into the electrolysis plant [MWh]
$e^{\mathrm{DC\_Elyzr}}_{bat,t}$	*, *	Electricity received in the electrolysis plant from discharging the battery [MWh]
$e^{\mathrm{DC\_Sell}}_{bat,t}$	*, *	Electricity discharged from the battery that is sold on the electricity market [MWh]
$e^{\mathrm{DC\_C}}_{bat,t}$	*, *	Electricity discharged from the battery that is used for pre-heating the syngas for FT synthesis [MWh]

$e_{bat,t}^{\mathrm{DC\_H}}$	*, *	Electricity discharged from the battery that is used for cooling down the syngas for storage [MWh]
$cost_t^{\mathrm{Grid}}$	*	Variable for cost of consuming electricity from the grid total [EUR2019]
$cost^{GridTot}$		Cost of consuming grid electricity averaged over the years of the optimization [EUR2019/yr]
l_t^{St1}		Indicator for loading and unloading 1 = loading, 0 = unloading (l in [0,1])
m_t^{FT}	*	Amount of syngas delivered to the FT synthesis facility [t/h]
m_t^{L}	*	Amount of syngas that is loaded in that operating hour [t/h]
m_t^{Un}	*	Amount of syngas that is unloaded in that operating hour [t/h]
c^{Sto}		Size in tonne of syngas storage [tonne]
sg_t^{St}	T	Mass of syngas stored in the storage system [tonnes]
sg_t^{Cycling}	*	Net amount of syngas that is stored or delivered in the operating hour [t/h]
q_t^{L}	*	Energy required for loading syngas [kW]
q_t^{Un}	*	Energy required for unloading syngas [kW]
q^{CapC}		Capacity of the cooler for loading syngas [kW]
q^{CapH}		Capacity of the heater for unloading syngas [kW]
$cost^{StoH}$		Storage heating cost [EUR2019/yr]
$cost^{\mathrm{StoC}}$		Storage cooling cost [EUR2019/yr]
$cost^{\mathrm{StoHC}}$		Cost for heating and cooling syngas to make it suitable for storage [EUR2019/yr]
$cost^{\mathrm{Sto}}$		Cost of storing syngas in a storage system [EUR2019/yr]
m_t^{Tot}	*	Total flow of syngas entering the FT synthesis plant [tonne/h]
z^{St1}		Cost - objective variable given a target of the total amount of syngas produced [EUR2019/yr]
rev_t^{Elec}	*	Total revenue of selling electricity [EUR2019]
rev^{Sell}		Revenue of selling electricity per year [EUR2019/yr]
z^{St2}		Net cost - objective variable of the second stage given a target of the total amount of syngas produced and revenue sold [EUR2019/yr]
l_t^{St2}	*	Binary for loading and unloading 1 = loading, 0 = unloading (l in [0,1])

$u_{bat,t}^{\mathrm{St2}}$	*, *	Binary for charging mode 0= discharging, 1= charging (u in [0,1])

Equations

Name	Domains	Description
$\mathrm{eqGenRES}_{res,t}$	*, *	The production of electricity per technology and timestep
eqGenCost		The electricity generation costs
$\mathrm{eqEleUse}_{t}$	*	The use of the generated electricity, charging, using in the electrolysis plant, curtailing, heating, cooling for storage, and selling to the grid for the grid electricity price [MWh]
$\mathrm{eqChargeInput}_{t}$	*	Energy input into the battery systems
$\mathrm{eqChargpUp}_{bat,t}$	*, *	Charging power upper bound
$\mathrm{eqDChargUp}_{bat,t}$	*, *	Discharging power upper bound
$\mathrm{eqEnergyNet}_{bat,t}$	*, *	Net hourly energy absorbed or injected energy in the battery
$\mathrm{eqEnergyBal}_{bat,t}$	*, *	Energy balance of the battery - Energy stored in the battery
$\mathrm{eqBatTight}_{bat,t}$	*, *	Tight formulation of the charging power and state of charge
$\mathrm{eqBatTightDC}_{bat,t}$	*, *	Tight formulation of the charging power and state of charge
$\mathrm{eqChargePowerLimit}_{bat,t}$	*BAT, T*	Charging power limit based on variable capacity
$\mathrm{leqDischargePowerLimit}_{bat,t}$	*BAT, T*	Discharging power limit based on variable capacity
$\mathrm{eqEnergyAdeq}_{bat,t}$	*, *	Tight formulation to ensure there is enough energy to discharge from the battery
$\mathrm{eqDCTight}_{bat,t}$	*, *	Tight formulation to ensure the discharge is smaller than the max charge capacity in a certain hour
$\mathrm{eqBat}_{bat,t}^{\mathrm{MinC}}$	*, *	Minimum SOC of the battery
$\mathrm{eqBat}_{bat,t}^{\mathrm{MaxC}}$	*, *	Maximum SOC of the battery
$\mathrm{eqRegBatCost}_{at}$	*	The regularized battery storage costs

eqTotBatCost		The total battery cost, summing different types of batteries (currently only Li-ion 4h)
eqBatCost2_{bat}	*	Calculates the actual battery storage costs without regulizers
$\text{eqDC}_{bat,t}^{\text{Bat}}$	*, *	The power discharged in each hour (energy), that is used for different applications within the plant
$\text{eqElyzr}_{t}^{\text{In}}$	*	Energy that is used by the electrolyzer from direct use, grid electricity, and the battery
eqElyzrCF_{t}	*	Link the energy generation of the renewables to the capacity factor of the electrolysis plant
eqProduction_{t}	*	Equation with the production amounts of syngas as a function of the capacity factor and the input capacity of the electrolysis plant
$\text{eqGrid}_{t}^{\text{Limit}}$	*	Maximum percentage of grid electricity used in each hour
$\text{eqGrid}_{t}^{\text{Cost}}$	*	The cost of consuming electricity from the grid
$\text{eqTot}^{\text{GridCost}}$		Cost of consuming grid electricity averaged over the years of the optimization
eqSGSupply_{t}	*	The syngas supply to the FT and the syngas storage from the electrolysis plant
eqLMin_{t}	*	Minimum loading of the syngas storage system
eqLMax_{t}	*	Maximum loading of the syngas storage system
eqLup_{t}	*	Upper bound unloading
eqUnup_{t}	*	Discharging power upper bound
eqCycleSG_{t}	*	Net hourly absorbed or injected syngas to the storage system
eqStSG_{t}	*	Storage balance
eqCyclicSto_{t}	*	Cyclic constraint on the storage mass
$\text{eqSto}_{t}^{\text{MinC}}$	*	Minimum amount of syngas in the storage system
$\text{eqSto}_{t}^{\text{MaxC}}$	*	Maximum amount of syngas in the storage system
$\text{eqQ}_{t}^{\text{L}}$	*	Equation for the energy required for loading

		the syngas into the storage system due to cooling requirements
eqQ_t^{Un}	*	Equation for the energy required for unloading syngas from the storage system due to pre-heating to FT conditions
$\text{eqQ}_t^{\text{CapL}}$	*	Cooling capacity needed for loading syngas into the storage system
$\text{eqQ}_t^{\text{CapUn}}$	*	Heating capacity needed for loading syngas into the storage system
eqSTO^{C}		Annualized CAPEX for investing into the cooler
eqSTO^{H}		Annualized CAPEXfor investing into the heater
eqStoElecH_t	*	Electricity requirement for operating the heater
eqStoElecC_t	*	Electricity requirement for operating the gas cooler
eqTotHC		Calculating the total storage heating and cooling cost
eqCostStoSG		The syngas storage cost, using the same regularized approach as for the battery
eqSGFT_t	*	Calculates the total flow of syngas entering the FT synthesis plant
eqSG		Syngas input into the SAF plant across the modeling horizon
eqUpCF		Upper capacity factor of the syngas electrolysis plant
eqObj		Objective function of the first stage objective, regarding the minimization of costs
eqSellCurt_t	*	Equation for selling in the second stage, the curtailed electricity in the first stage, while re-optimizing the operation
eqTotSellRev		Total revenue from selling electricity
eqObj2		Second stage objective
eqLup2_t	*	Charging power upper bound
eqUnup2_t	*	Discharging power upper bound

$\text{eqChargpup2}_{bat,t}$	*, *	Charging power upper bound
$\text{eqDChargplup2}_{bat,t}$	*, *	Discharging power upper bound

Naming conventions

Throughout the mathematical formulation, variables are represented in lowercase italics, and parameters are capitalized in italics. The domain of a variable is displayed in lowercase italic subscript, while the roman superscript provides additional details about the symbols used in the model. The sets after for all are written capitalized.

Equation Definitions

Eq. (1) describes the amount of electricity produced by the installed capacity of renewables c_{res}^{RES}, over the capacity factor input parameter across the years from 2019 – 2024 ($CF_{res,t,r}^{\text{Elec}}$). This is summed over the region, which is exclusively the Netherlands in this work. This results in the hourly electricity produced by the generation assets of set RES (onshore wind, offshore wind and solar PV).

$$\textbf{eqGenRES}_{res,t} \qquad gen_{res,t}^{\text{In}} = \sum_{r} \left(CF_{res,t,r}^{\text{Elec}} \cdot c_{res}^{\text{RES}} \right) \qquad \forall\ RES, T \qquad (1)$$

The total cost of generation are based on several aspects:

1. Sum of investment cost based on the installed capacity of generation in MW. Parameter RES_{res}^{Fixed} contains the capacity-based annualized investment cost of the renewable generation.
2. Sum of OPEX cost based on the amount of electricity produced in MWh, $OPEX_{res,scen}^{\text{RES}}$ contains the variable cost of the operation.
3. Grid tariffs (GRD^{Own}), cost of consuming self generated electricity.

The OPEX and grid tariff category is divided over the number of years considered in the optimization. Since both the OPEX and annualized capex are considered on a yearly basis.

$$\textbf{eqGenCost} \qquad cost^{Gen} = \sum_{res} \left(c_{res}^{\text{RES}} \cdot RES_{res}^{\text{Fixed}} \cdot 1000 \right) + \frac{\sum_{res,t} \left(gen_{res,t}^{\text{In}} \cdot \sum_{scen} OPEX_{res,scen}^{\text{RES}} \right) + \sum_{res,t} \left(gen_{res,t}^{\text{In}} \cdot GRD^{\text{Own}} \right)}{YR} \qquad (2)$$

The electricity produced via the generation assets is used in a range of different applications, namely: electrolyzer (e_t^{Elyzr}), for cooling of syngas before temporal storage (e_t^{C}), reheating after syngas storage (e_t^{H}), charging the battery (e_t^{Bat}), curtailing the produced electricity (e_t^{Curt}), see Eq. (3). This variable is set for each hour in the optimization via set T.

$$\textbf{eqEleUse}_t \qquad \sum_{res} gen^{\text{In}}_{res,t} = e^{\text{Elyzr}}_t + e^{\text{Curt}}_t + e^{\text{Bat}}_t + e^{\text{H}}_t + e^{\text{C}}_t \qquad \forall\ T \quad (3)$$

Eqs. (4) – (20) describe the battery energy flows, charging, discharging, self-discharge, and cost of the battery unit operation in the optimization. In Eq. (4), the renewable electricity entering the battery equals the battery's charging power. The set BAT contains the different battery technologies that might be applied in the system. In this work, specifically, a 4-hour Li-ion battery was considered. The charging capacity is measured in MW, and since an hourly model was considered, this equates to electricity production in MWh.

$$\textbf{eqChargeInput}_t \qquad e^{\text{Bat}}_t = \sum_{bat} p^{\text{C}}_{bat,t} \qquad \forall\ T \quad (4)$$

The maximum charging capacity in Eq. (5) is less than or equal to the C-rate of the battery (1/4 hours - C^{Max}_{bat}), and the maximum power of the battery (P^{Max}) that can be charged/discharged. $u_{bat,t}$ is the charging/discharging indicator, where 0 = discharging, 1 = charging. A parallel equation is in place for discharging in Eq. (6).

$$\textbf{eqChargpUp}_{bat,t} \qquad p^{\text{C}}_{bat,t} \leq C^{\text{Max}}_{bat} \cdot P^{Max} \cdot u_{bat,t} \qquad \forall\ BAT, T \quad (5)$$

$$\textbf{eqDChargUp}_{bat,t} \qquad p^{\text{DC}}_{bat,t} \leq C^{\text{Max}}_{bat} \cdot P^{Max} \cdot (1 - u_{bat,t}) \qquad \forall\ BAT, T \quad (6)$$

In Eq. (7), the (net) energy which enters or leaves the battery ($e^{\text{Net}}_{bat,t}$) is calculated, taking into account that there are charging (ETA^{C}) and discharging (ETA^{DC}) losses. This variable can be positive when the battery is being charged and negative when it is being discharged. The state of charge (SOC, $e^{\text{SOC}}_{bat,t}$) of the battery capacity is calculated using Eq. (8), where the net energy input is added to the SOC from the previous hour. The SOC for the previous hour is corrected by the battery's self-discharge.

$$\textbf{eqEnergyNet}_{bat,t} \qquad e^{\text{Net}}_{bat,t} = ETA^{\text{C}} \cdot p^{\text{C}}_{bat,t} - \frac{p^{\text{DC}}_{bat,t}}{ETA^{DC}} \qquad \forall\ BAT, T \quad (7)$$

$$\textbf{eqEnergyBal}_{bat,t} \qquad e^{\text{SOC}}_{bat,t--1} \cdot (1 - SELF^{DC}) + e^{\text{Net}}_{bat,t} = e^{\text{SOC}}_{bat,t} \qquad \forall\ BAT, T \quad (8)$$

Eqs. (9) and (10) are introduced to ensure the battery description is formulated tightly, thereby improving solver performance. By introducing these additional conditions, the solver can find a tighter lower bound more easily (Chen & Baldick, 2022).

eqBatTight$_{bat,t}$ $$p_{bat,t}^{\mathrm{C}} \leq \frac{P^{\mathrm{Max}} \cdot C_{bat}^{\mathrm{Max}}}{ETA^{\mathrm{C}}} \quad \forall\, BAT,T \qquad (9)$$

eqBatTightDC$_{bat,t}$ $$p_{bat,t}^{\mathrm{DC}} \leq (P^{\mathrm{Max}} \cdot C_{bat}^{\mathrm{Max}} - e_{bat,t--1}^{\mathrm{SOC}}) \cdot ETA^{\mathrm{DC}} \quad \forall\, BAT,T \qquad (10)$$

In Eqs. (11) and (12), the maximum charging and discharging limits are specified via the C-rate, charging/discharging efficiency, and capacity of the battery.

eqChargePowerLimit$_{bat,t}$ $$p_{bat,t}^{\mathrm{C}} \leq \frac{C_{bat}^{\mathrm{max}}}{ETA^{\mathrm{C}}} \cdot c_{bat}^{\mathrm{Bat}} \quad \forall\, BAT,T \qquad (11)$$

eqDischargePowerLimit$_{bat,t}$ $$p_{bat,t}^{\mathrm{DC}} \leq C_{bat}^{\mathrm{Max}} \cdot c_{bat}^{\mathrm{Bat}} \cdot ETA^{DC} \quad \forall\, BAT,T \qquad (12)$$

Eq. (13) is an adequacy constraint on the discharge, where the SOC of the previous hour is checked to be sufficient for discharging in the current hour.

eqEnergyAdeq$_{bat,t}$ $$p_{bat,t}^{\mathrm{DC}} \leq e_{bat,t--1}^{\mathrm{SOC}} \cdot ETA^{\mathrm{DC}} \quad \forall\, BAT,T \qquad (13)$$

eqDCTight$_{bat,t}$ $$p_{bat,t}^{\mathrm{DC}} \leq P^{Max} - p_{bat,t}^{\mathrm{C}} \quad \forall\, BAT,T \qquad (14)$$

The battery can be charged to a maximum state of charge of 95% and a minimum SOC of 5% (via SOC^{Max} and SOC^{Min}) for safety and longevity reasons, which is captured in Eqs. (15) and (16).

eqBat$_{bat,t}^{\mathrm{MinC}}$ $$e_{bat,t}^{\mathrm{SOC}} \leq c_{bat}^{\mathrm{Bat}} \cdot SOC^{Max} \quad \forall\, BAT,T \qquad (15)$$

eqBat$_{bat,t}^{\mathrm{MaxC}}$ $$e_{bat,t}^{\mathrm{SOC}} \geq c_{bat}^{\mathrm{Bat}} \cdot SOC^{Min} \quad \forall\, BAT,T \qquad (16)$$

In Eq. (17), RG^{C} and RG^{DC} are regularizers for charging and discharging respectively. The concept for these regularizers is described in the main text of the current work. Via the battery cost (reg_{bat}^{Bat}) the penalties on simultaneous charging and discharging are part of the objective function. Moreover, the capacity of the battery (c_{bat}^{Bat}), is multiplied by the annualized battery cost ($BAT_{bat}^{\mathrm{CAPEX}}$) to find the cost, including the regularizer penalty, for the different battery types in the model.

eqRegBatCost$_{bat}$ $$reg_{bat}^{\mathrm{Bat}} = c_{bat}^{\mathrm{Bat}} \cdot BAT_{bat}^{\mathrm{CAPEX}} + \sum_{t} (RG^{\mathrm{C}} \cdot p_{bat,t}^{\mathrm{C}} + RG^{\mathrm{DC}} \cdot p_{bat,t}^{\mathrm{DC}}) \quad \forall\, BAT \qquad (17)$$

The RG^{C} and RG^{DC} need to be chosen such that they fulfill the condition in Eq. (18),

following Han et al., (2024). In order to guarantee this condition, the regularizer values are calculated by using Eqs. (19) and (20). This combination ensured that the condition in Eq. (18) was satisfied, so the regularizers achieved the desired goal of accurately describing battery behavior without introducing binary variables.

RegularizerCondition $$RG^{C} + {ETA^{DC}}^{2} \cdot RG^{\mathrm{DC}} \geq 1 - {ETA^{DC}}^{2} \quad (18)$$

RegularizedCharging $$RG^{C} = \frac{1-(ETA^{DC})^{2}}{1+(ETA^{DC})^{2}} \quad (19)$$

RegularizedDischarging $$RG^{DC} = RG^{C} + \frac{1-(ETA^{C})^{2}}{1+(ETA^{C})^{2}} \cdot \frac{1}{5} \quad (20)$$

By summing all the different battery technologies, the total cost is given in Eq. (21). Alternatively, the costs are calculated without the regularized penalty in Eq. (22); this value is used in the final reporting of the results.

eqTotBatCost $$cost^{RegBat} = \sum_{bat} reg_{bat}^{\mathrm{Bat}} \quad (21)$$

eqBatCost2$_{bat}$ $$cost_{bat}^{\mathrm{Bat}} = c_{bat}^{\mathrm{Bat}} \cdot BAT_{bat}^{\mathrm{CAPEX}} \qquad \forall\, BAT \quad (22)$$

When the battery discharges (Eq. (23)), the electricity can be used by different applications, namely: selling to the grid (only in stage 2, $e_{bat,t}^{\mathrm{DC\_Sell}}$), used in the electrolyzer ($e_{bat,t}^{\mathrm{DC\_Elyzr}}$), re-heating syngas ($e_{bat,t}^{\mathrm{DC\_H}}$), cooling syngas upon storage ($e_{bat,t}^{\mathrm{DC\_C}}$).

eqDC$_{bat,t}^{\mathbf{Bat}}$ $$p_{bat,t}^{\mathrm{DC}} = e_{bat,t}^{\mathrm{DC\_Sell}} + e_{bat,t}^{\mathrm{DC\_Elyzr}} + e_{bat,t}^{\mathrm{DC\_H}} + e_{bat,t}^{\mathrm{DC\_H}} \qquad \forall\, BAT, T \quad (23)$$

The electricity from discharging the battery, which is consumed from the grid (e_{t}^{Grid}) and which is produced in the dedicated generation assets (e_{t}^{Elyzr}) serves as the input to the electrolyzer (e_{t}^{Input}), in Eq. (24).

eqElyzr$_{t}^{\mathbf{In}}$ $$e_{t}^{\mathrm{Elyzr}} + \sum_{bat} e_{bat,t}^{\mathrm{DC\_Elyzr}} + e_{t}^{\mathrm{Grid}} = e_{t}^{\mathrm{Input}} \qquad \forall\, T \quad (24)$$

This electricity input equals the capacity parameter multiplied by the capacity factor, which can be set for each hour of the electrolyzer, as given in Eq. (25).

eqElyzrCF$_{t}$ $$e_{t}^{\mathrm{Input}} = cf_{t}^{\mathrm{Elyzr}} \cdot C^{\mathrm{Elyzr}} \qquad \forall\, T \quad (25)$$

Using the linearized hot-box submodel, the electrolyzer's capacity factor is linked to syngas production each hour. The gradient (A^{Elyzr}) and intercept (B^{Elyzr}) result in a

capacity-independent hourly production. m_t^{SG} is the hourly syngas production in tonnes, in Eq. (26).

eqProduction$_t$ $$m_t^{\mathrm{SG}} = \frac{C^{Elyzr} \cdot (A^{Elyzr} \cdot cf_t^{\mathrm{Elyzr}} \cdot 100 - B^{Elyzr})}{1000} \quad \forall T \quad (26)$$

The amount of electricity that can be consumed from the grid (e_t^{Grid}) is practically constrained by the background emissions of the syngas production supply chain, in Eq. (27), this is described in detail in the main text.

eqGrid$_t^{\mathrm{Limit}}$ $$e_t^{\mathrm{Grid}} \leq \frac{GE^{\mathrm{Cons}}}{100} \cdot e_t^{\mathrm{Input}} \quad \forall T \quad (27)$$

The electricity cost in Eq. (28) consists of two factors: variable electricity prices and fixed grid tariffs for purchasing electricity from the grid. The result is the cost of electricity from grid consumption. To account for the yearly nature of the model over the 6-year horizon, the electricity costs are averaged over the years (YR) in Eq. (29).

eqGrid$_t^{\mathrm{Cost}}$ $$e_t^{\mathrm{Grid}} \cdot ELEC_t^{\mathrm{Price}} + e_t^{\mathrm{Grid}} \cdot GRD^{\mathrm{Buy}} = cost_t^{\mathrm{Grid}} \quad \forall T \quad (28)$$

eqTot^{GridCost} $$\frac{\sum_t cost_t^{\mathrm{Grid}}}{YR} = cost^{GridTot} \quad (29)$$

The produced syngas from the electrolyzer can either be used directly (m_t^{FT}) or stored in the pipeline storage system (m_t^{L}), Eq. (30). Since it involves a storage system, the same modeling structure is applied as with the battery previously described.

eqSGSupply$_t$ $$m_t^{\mathrm{SG}} = m_t^{\mathrm{FT}} + m_t^{\mathrm{L}} \quad \forall T \quad (30)$$

The sg_t^{Cycling} is the amount of syngas that is loaded or unloaded in the system storage in a specific hour, following Eq. (31).

eqCycleSG$_t$ $$sg_t^{\mathrm{Cycling}} = m_t^{\mathrm{L}} - m_t^{\mathrm{Un}} \quad \forall T \quad (31)$$

The variable is positive upon loading and negative upon unloading. This variable is constrained by the maximum loading and unloading capacity ST^{MaxL} in tonnes/hour via Eq. (32) and (33).

eqLMin$_t$ $$-ST^{MaxL} \leq sg_t^{\mathrm{Cycling}} \quad \forall T \quad (32)$$

eqLMax$_t$ $$sg_t^{\mathrm{Cycling}} \leq ST^{MaxL} \quad \forall T \quad (33)$$

l_t is the continuous variable from 0 to 1 that indicates the loading and unloading state

for Eqs. (34) and (35).

eqLup$_t$ $$m_t^{\mathrm{L}} \leq ST^{MaxL} \cdot l_t \qquad \forall\, T \qquad (34)$$

eqUnup$_t$ $$m_t^{\mathrm{Un}} \leq ST^{MaxL} \cdot (1 - l_t) \qquad \forall\, T \qquad (35)$$

The sg_t^{St} is the storage level of the syngas storage system and consists of the storage level in the previous hour sg_{t--1}^{St} and the net amount, which is added or unloaded sg_t^{Cycling} in Eq. (36). Eq. (37) is a cyclic constraint to ensure that the storage level is the same in the first period as the last period. This simulates that the system will be in use after the optimization horizon.

eqStSG$_t$ $$sg_{t--1}^{\mathrm{St}} + sg_t^{\mathrm{Cycling}} = sg_t^{\mathrm{St}} \qquad \forall\, T \qquad (36)$$

eqCyclicSto$_t$ $$sg_{t=0}^{\mathrm{St}} = sg_{t=n}^{\mathrm{St}} \qquad \forall\, T \qquad (37)$$

Eq. (38) and (39) are the min/max storage volume constraints, which are set by the design capacity c^{Sto}.

eqSto$_t^{\mathrm{MinC}}$ $$sg_t^{\mathrm{St}} \leq c^{Sto} \cdot SOC^{Max} \qquad \forall\, T \qquad (38)$$

eqSto$_t^{\mathrm{MaxC}}$ $$sg_t^{\mathrm{St}} \geq c^{Sto} \cdot SOC^{Min} \qquad \forall\, T \qquad (39)$$

For safe storage, the syngas needs to be cooled down and reheated; the amount of heat input is calculated following Eqs. (40) and (41). CPM^{SG} is the heat capacity of the syngas, while D^t is the temperature that needs to be cooled and re-heated.

eqQ$_t^{\mathrm{L}}$ $$q_t^{\mathrm{L}} = \frac{\frac{m_t^{\mathrm{L}} \cdot CPM^{SG} \cdot D^t}{1000}}{3600} \qquad \forall\, T \qquad (40)$$

eqQ$_t^{\mathrm{Un}}$ $$q_t^{\mathrm{Un}} = \frac{\frac{m_t^{\mathrm{Un}} \cdot CPM^{SG} \cdot D^t}{1000}}{3600} \qquad \forall\, T \qquad (41)$$

The actual heating and cooling capacity is set via separate variables (q^{CapC}) and (q^{CapH}), in Eqs. (42) and (43).

eqQ$_t^{\mathrm{CapL}}$ $$q^{\mathrm{CapC}} \geq q_t^{\mathrm{L}} \qquad \forall\, T \qquad (42)$$

$$\mathbf{eqQ_t^{CapUn}} \qquad q^{\mathrm{CapH}} \geq q_t^{\mathrm{Un}} \qquad \forall\, T \quad (43)$$

These design heat and cooling capacities are used to calculate the investment cost of the cooler and heater, $CAPEX^{\mathrm{Cooler}}$ and $CAPEX^{\mathrm{H}eater}$ respectively. Since these CAPEX are not yet annualized, the CRF^{Sto} is used to find the annualized investment cost of heating $cost^{StoH}$ and cooling $Cost^{StoC}$ via Eqs. (44) and (45). Later in Eq. (48), these investment costs are summed to find the investment cost of the heating and cooling system for syngas storage.

$$\mathbf{eqSTO^{C}} \qquad q^{\mathrm{CapC}} \cdot CAPEX^{\mathrm{Cooler}} \cdot CRF^{Sto} = cost^{StoH} \qquad (44)$$

$$\mathbf{eqSTO^{H}} \qquad q^{\mathrm{CapH}} \cdot CAPEX^{\mathrm{Heater}} \cdot CRF^{\mathrm{Sto}} = cost^{\mathrm{StoC}} \qquad (45)$$

The heating and cooling systems were assumed to be powered by electricity from the battery or heating system. With the COP for heating and cooling, the required electricity is determined using Eqs. (46) and (47).

$$\mathbf{eqStoElecH_t} \qquad \frac{q_t^{\mathrm{Un}}}{COP^{\mathrm{Heater}}} = \sum_{bat} e_{bat,t}^{\mathrm{DC\_h}} + e_t^{\mathrm{H}} \qquad \forall\, T \quad (46)$$

$$\mathbf{eqStoElecC_t} \qquad \frac{q_t^{\mathrm{L}}}{COP^{\mathrm{Cooler}}} = \sum_{bat} e_{bat,t}^{\mathrm{DC\_c}} + e_t^{\mathrm{C}} \qquad \forall\, T \quad (47)$$

$$\mathbf{eqTotHC} \qquad cost^{StoHC} = cost^{\mathrm{StoH}} + cost^{\mathrm{StoC}} \qquad (48)$$

The total cost for syngas storage comprises the storage system and the cost of heating and cooling. In Eq. (49), the regularizers are also included for the syngas storage system to prevent loading and unloading. Simultaneous loading and unloading can occur in a tank storage system, but because it requires an additional cooling and heating cycle, it is undesirable. Therefore, the same approach was applied as in the battery storage system.

$$\mathbf{eqCostStoSG} \qquad cost^{\mathrm{StoReg}} = c^{\mathrm{Sto}} \cdot SG^{\mathrm{Storage}} + cost^{\mathrm{StoHC}} + \sum_t (RG^{\mathrm{C}} \cdot q_t^{\mathrm{L}} + RG^{\mathrm{DC}} \cdot q_t^{\mathrm{Un}}) \qquad (49)$$

In Eq. (50), the syngas from the storage system (m_t^{Un}) and which is directly passed on from the electrolyzer to the FT synthesis plant (m_t^{FT}) make up the total amount of syngas which needs to be consumed in the FT plant.

eqSGFT$_t$ $$m_t^{\mathrm{Tot}} = m_t^{\mathrm{FT}} + m_t^{\mathrm{Un}} \quad \forall T \quad (50)$$

The $SG^{\mathrm{Prod}}$the yearly syngas production target in ktonnes per year, corrected for the number of years, needs to be equal to the total amount of syngas entering the FT plant (See Eq. (51)). This is the target in the optimization that needs to be matched, which is set using the average capacity factor across the year.

eqSG $$SG^{\mathrm{Prod}} \cdot YR = \sum_t \left(\frac{m_t^{\mathrm{Tot}}}{1000}\right) \quad (51)$$

z^{St1} is the objective function of the first stage, comprised of the total cost of grid consumed electricity ($Cost^{\mathrm{GridTot}}$), storage of syngas ($cost^{\mathrm{StoReg}}$) and batteries ($cost^{\mathrm{RegBat}}$) and generation ($cost^{\mathrm{Gen}}$), in Eq. (52).

eqObj $$cost^{\mathrm{Gen}} + cost^{\mathrm{RegBat}} + cost^{\mathrm{StoReg}} + Cost^{\mathrm{GridTot}} = z^{\mathrm{St1}} \quad (52)$$

In the second stage, the capacity sizing decisions are fixed, and the curtailed electricity can be sold (e_t^{Curt}) and electricity can be sold directly back to the grid ($e_{bat,t}^{\mathrm{DC\_sell}}$) for the prevailing electricity price ($ELEC_t^{\mathrm{Price}}$). The annual revenue (rev^{Sell}) is found using Eq. (54).

eqSellCurt$_t$ $$\left(e_t^{\mathrm{Curt}} + \sum_{bat} e_{bat,t}^{\mathrm{DC\_sell}}\right) \cdot ELEC_t^{\mathrm{Price}} = rev_t^{\mathrm{Elec}} \quad \forall T \quad (53)$$

eqTotSellRev $$\frac{\sum_t rev_t^{\mathrm{Elec}}}{YR} = rev^{\mathrm{Sell}} \quad (54)$$

From the total cost in stage 1, these revenues are subtracted to find the minimum cost of producing a fixed quantity of syngas, given that all revenues from the electricity system reduce the cost of syngas production, as shown in Eq. (55).

eqObj2 $$z^{\mathrm{St1}} - rev^{\mathrm{Sell}} = z^{\mathrm{St2}} \quad (55)$$

Eqs. (56) – (59) are similar to the loading/unloading and charging/discharging equations covered in Eqs. (34), (35), (5), and (6), respectively. The difference in these equations is the definition of l_t^{St2} and $u_{bat,t}^{\mathrm{St2}}$. In the first stage, the indicator for loading and unloading, charging and discharging is a continuous variable ranging from 0 to 1. At this stage, it is strictly a binary variable to ensure the battery's operational behavior is described correctly.

eqLup2$_t$ $$m_t^{\mathrm{L}} \leq ST^{MaxL} \cdot l_t^{\mathrm{St2}} \quad \forall T \quad (56)$$

eqUnup2$_t$ $m_t^{\text{Un}} \leq ST^{MaxL} \cdot (1 - l_t^{\text{St2}})$ $\forall\, T$ (57)

eqChargpup2$_{bat,t}$ $p_{bat,t}^{\text{C}} \leq C_{bat}^{\text{Max}} \cdot P^{Max} \cdot u_{bat,t}^{\text{St2}}$ $\forall\, BAT, T$ (58)

eqDChargplup2$_{bat,t}$ $p_{bat,t}^{\text{DC}} \leq C_{bat}^{\text{Max}} \cdot P^{Max} \cdot (1 - u_{bat,t}^{\text{St2}})$ $\forall\, BAT, T$ (59)

Variable declarations

${gen^{\text{In}}}_{*,*} \geq 0\ \forall\ *,*$

$c_*^{Res} \geq 0\ \forall\ *$

$cost^{\text{Gen}} \geq 0\ \forall$

$e_*^{Elyz\text{r}} \geq 0\ \forall\ *$

$e_*^{\text{Curt}} \geq 0\ \forall\ *$

$e_*^{\text{Bat}} \geq 0\ \forall\ *$

$e_*^{\text{H}} \geq 0\ \forall\ *$

$e_*^{\text{C}} \geq 0\ \forall\ *$

$p_{bat,t}^{\text{Bat}} \geq 0\ \forall\ BAT, T$

$p_{bat,t}^{\text{DC}} \geq 0\ \forall\ BAT, T$

$e_{bat,t}^{SOC} \geq 0\ \forall\ BAT, T$

$c_*^{\text{Bat}} \geq 0\ \forall\ *$

$reg_{bat}^{Bat} \geq 0\ \forall\ BAT$

$cost^{RegBat} \geq 0\ \forall$

$cost_{bat}^{\text{Bat}} \geq 0\ \forall\ BAT$

$e_{*,*}^{\text{DC\_Sell}} \geq 0\ \forall\ *,*$

$e_{*,*}^{\text{DC\_Elyzr}} \geq 0\ \forall\ *,*$

$e_{*,*}^{DC\_\text{H}} \geq 0\ \forall\ *,*$

$e_{*,*}^{DC\_C} \geq 0\ \forall\ *,*$

$e_*^{\text{Grid}} \geq 0\ \forall\ *$

$e_*^{\text{Input}} \geq 0\ \forall\ *$

$cf_*^{\text{Elyzr}} \geq 0\ \forall\ *$

$m_*^{SG} \geq 0\ \forall\ *$

$m_*^{FT} \geq 0\ \forall\ *$

$m_*^{L} \geq 0\ \forall\ *$

$m_*^{\text{Un}} \geq 0\ \forall\ *$

$sg_t^{\text{St}} \geq 0\ \forall\ T$

$c^{\text{Sto}} \geq 0\ \forall$

$q_*^{l} \geq 0\ \forall\ *$

$q_*^{\text{Un}} \geq 0\ \forall\ *$

$q^{\text{CapC}} \geq 0\ \forall$

$q^{\text{CapH}} \geq 0\ \forall$

$cost^{\text{StoH}} \geq 0\ \forall$

$cost^{\text{StoC}} \geq 0\ \forall$
$cost^{\text{StoHC}} \geq 0\ \forall$
$cost^{\text{Sto}} \geq 0\ \forall$
$m_{*}^{\text{Tot}} \geq 0\ \forall\ *$
$l_{*}^{\text{St2}} \in 0{,}1\ \forall\ *$
$u_{*,*}^{\text{St2}} \in 0{,}1\ \forall\ *{,}*$

References

Adriaansens, M., & Jetten, R. (2021). Kabinetsaanpak Klimaatbeleid Voorzienings- en leveringszekerheid energie. In *Tweede Kamer der Staten-Generaal: Vol. 32 851* (Issue 71, pp. 1–8). https://www.tweedekamer.nl/kamerstukken/moties/detail?id=2021D47333&did=2021D47333

Agora Energiewende. (2022). *Power-2-Heat* (Issue December 20, 2016). http://www.voltachem.com/research/power-2-heat

Chen, Y., & Baldick, R. (2022). Battery Storage Formulation and Impact on Day Ahead Security Constrained Unit Commitment. *IEEE Transactions on Power Systems*, *37*(5), 3995–4005. https://doi.org/10.1109/TPWRS.2022.3144241

Danish Energy Agency. (2025). *Technology Data for Generation of Electricity and District Heating*. https://ens.dk/en/analyses-and-statistics/technology-data-generation-electricity-and-district-heating

ENTSO-E. (2025). *ENTSO-E Transparency Platform: Day-Ahead Prices*. ENTSO-E. https://transparency.entsoe.eu/transmission-domain/r2/dayAheadPrices/show

Fraunhofer IEG. (2024). *Cooling Technologies, Market, Best Practices and Planning in Europe*.

Han, D., Jiang, N., Dey, S. S., & Xie, W. (2024). Regularized MIP Model for Optimal Power Flow with Energy Storage Systems and its Applications. *ArXiv Preprint ArXiv:2402.04406*, 1–39.

Papadias, D. D., & Ahluwalia, R. K. (2021). Bulk storage of hydrogen. *International Journal of Hydrogen Energy*, *46*(70), 34527–34541. https://doi.org/10.1016/j.ijhydene.2021.08.028

Rangelova, K., & Jones, D. (2025). *Solar electricity every hour of every day is here and it changes everything* (Issue June).
